# Supplementary material for: Characterization of protein unfolding by fast cross-linking mass spectrometry using di-ortho-phthalaldehyde cross-linkers
Source: Nat Commun. 2022 Mar 18;13:1468. doi: 10.1038/s41467-022-28879-4 (PMC8933431; doi:10.1038/s41467-022-28879-4)
Supplement: Supplementary file 1 — Supplementary Information [file 41467_2022_28879_MOESM1_ESM.pdf]

Supplementary Information

Characterization of protein unfolding by fast cross-linking mass spectrometry using di-*ortho*-phthalaldehyde cross-linkers

Jian-Hua Wang[#,1,2], Yu-Liang Tang[#,3], Zhou Gong[4], Rohit Jain[5], Fan Xiao[3], Yu Zhou[1,2], Dan Tan[1], Qiang Li[3], Niu Huang[1,2], Shu-Qun Liu[6], Keqiong Ye[7,8], Chun Tang[*,3,4], Meng-Qiu Dong[*,1,2], Xiaoguang Lei[*,3]

[1]National Institute of Biological Sciences (NIBS), Beijing 102206, China; [2]Tsinghua Institute of Multidisciplinary Biomedical Research, Tsinghua University, Beijing 102206, China; [3]Beijing National Laboratory for Molecular Sciences, Key Laboratory of Bioorganic Chemistry and Molecular Engineering of Ministry of Education, Synthetic and Functional Biomolecules Center, College of Chemistry and Molecular Engineering, Peking-Tsinghua Center for Life Science, Peking University, Beijing 100871, China; [4]Innovation Academy for Precision Measurement Science and Technology, Chinese Academy of Sciences, Wuhan, Hubei 430071, China; [5]University of Massachusetts Medical School, Worcester MA 01605, USA; [6]State Key Laboratory for Conservation and Utilization of Bio-Resources in Yunnan, Yunnan University, Kunming 650091, Yunnan, China; [7]Key Laboratory of RNA Biology, CAS Center for Excellence in Biomacromolecules, Institute of Biophysics, Chinese Academy of Sciences, Beijing 100101, China; [8]University of Chinese Academy of Sciences, Beijing 100049, China

[#] Equal contribution
[*] Co-corresponding authors
E-mail: Tang_Chun@pku.edu.cn; dongmengqiu@nibs.ac.cn; xglei@pku.edu.cn

Contents

Supplementary Figure 1. Optimization of cross-linking conditions using BSA. 3
Supplementary Figure 2. Rate kinetics of OPA and NHS ester with Boc-OMe-lysine. 4
Supplementary Figure 3. FRET-based system comparison of the reaction rate of Cy5-OPA and Cy5-NHS ester with Cy3-K peptide. 5
Supplementary Figure 4. Comparing the rates of cross-linking of DOPA2 and DSS based on an EGTA-resistant FRET signal of Yellow Cameleon 3.6 (YC3.6). 6
Supplementary Figure 5. A broader comparison between DOPA2 and other cross-linkers. 7
Supplementary Figure 6. DOPA2 cross-linking of BSA was quenched by hydrazine within five seconds. .8
Supplementary Figure 7. Comparing DOPA2 or DSS cross-linking reactions of different durations. 9
Supplementary Figure 8. Annotated MS/MS spectrum of the DOPA2-linked inter-molecular peptide pair from BSA. 10
Supplementary Figure 9. The four unclassified cross-links of BSA by DOPA2 cross-linking in urea. 11
Supplementary Figure 10. Analysis of the unfolding process of RNase A in urea using DOPA2 under reduction conditions...12
Supplementary Figure 11. Scatter plot of DOPA2 cross-links on the primary sequence of BSA. 13
Supplementary Figure 12. Scatter plots of DOPA2 cross-links on the primary sequences of SNase and RNase A. 14
Supplementary Figure 13. Distribution of the amino acid distances between two linked residues of the native versus the non-native clusters of DOPA2 cross-links. 15
Supplementary Figure 14. The Cα-Cα distance distribution of DOPA2 obtained by MD simulations.......16
Supplementary Table 1. Peptides tested for OPA selectivity. 17
Supplementary Table 2. Products of ten synthesized peptides and OPA. 18
Supplementary Table 3. The composition of the ten-protein mixture and the PDB code of the proteins in Figure 3d. 19
Supplementary Table 4. The intensity of different products in peptide samples cross-linked by DOPA2 or DSS at low pH and in the presence of denaturants. 20
^{1}H NMR and ^{13}C NMR spectra 21
Supplementary Methods 27

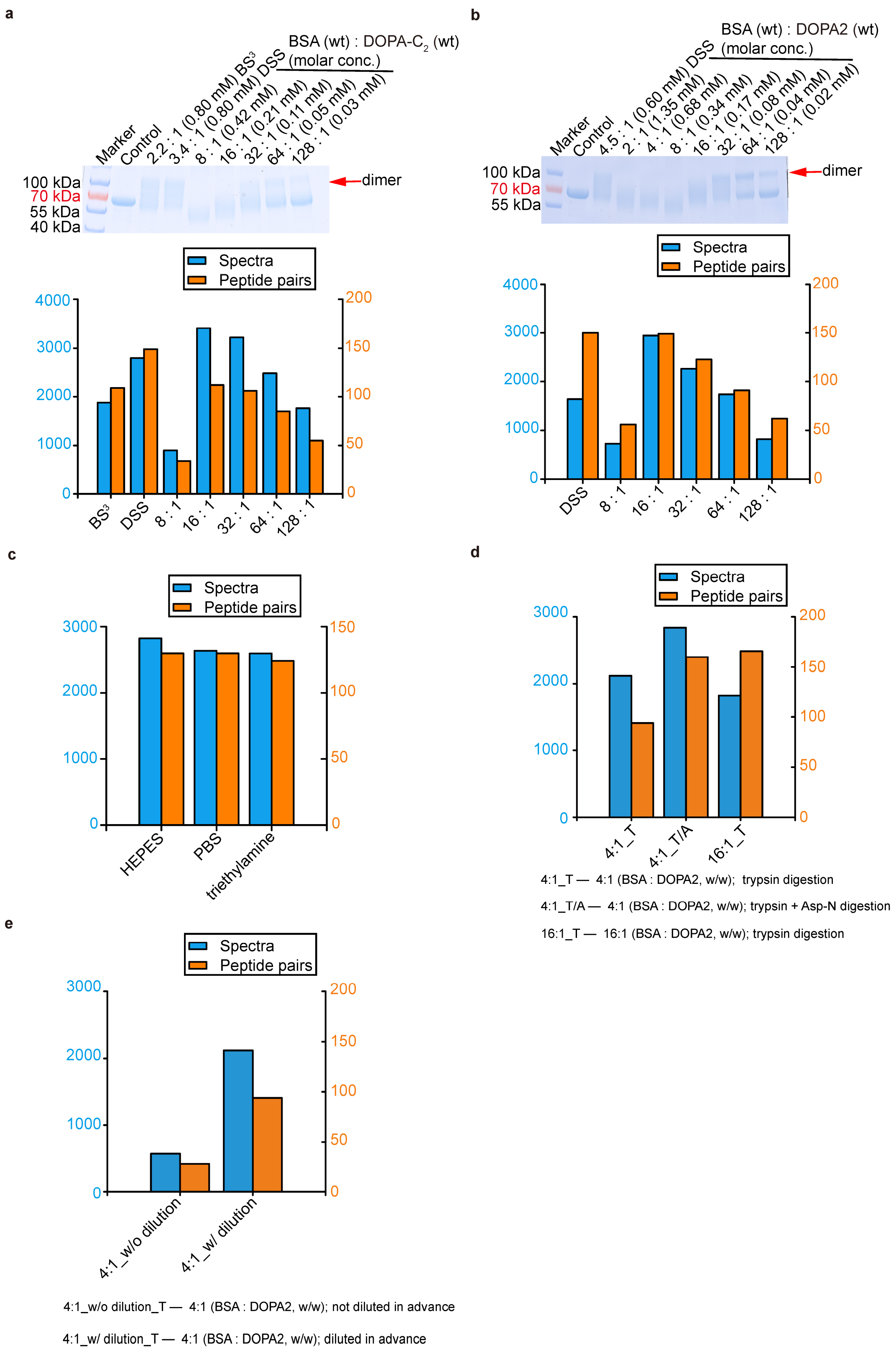

Supplementary Figure 1. Optimization of cross-linking conditions using BSA.

(a-b) Optimization of the working concentration of cross-linkers DOPA-C_2 and DOPA2. (c) Performance of DOPA2 in HEPES buffer, PBS buffer, and triethylamine buffer on BSA. (d) Comparison of cross-linking effects of DOPA2 by digestion with trypsin alone or with trypsin plus Asp-N. (e) Comparison of cross-linking effects of DOPA2 with or without pre-dilution to 2× the final concentration. Identified cross-links were filtered by requiring FDR < 0.01 at the spectral level. One cross-linking experiment was performed for each protein sample in a-e. Source data for a-e are provided as a Source Data file.

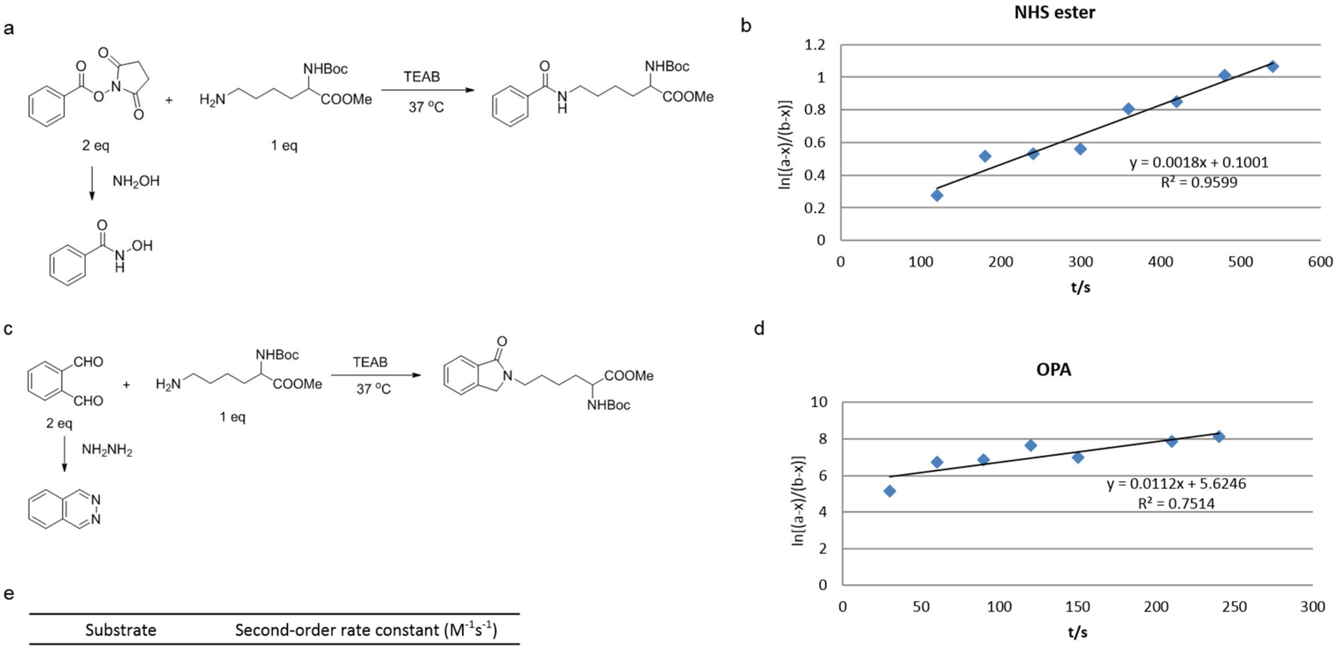

e

Substrate	Second-order rate constant ($M^{-1}s^{-1}$)
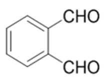	2.24
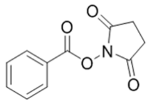	0.36

Supplementary Figure 2. Rate kinetics of OPA and NHS ester with Boc-OMe-lysine.

(a) The reaction of NHS ester with lysine afforded the amide and the excessive NHS esters were quenched by hydroxylamine. (b) The rate curve of the reaction of NHS ester with lysine. (c) The reaction of OPA with lysine afforded the phthalimidine and the excessive OPA was quenched by hydrazine to yield phthalazine. (d) The rate curve of the reaction of OPA with lysine. (e) The second-order rate constant of OPA and NHS ester with lysine. Source data for b and d are provided as a Source Data file.

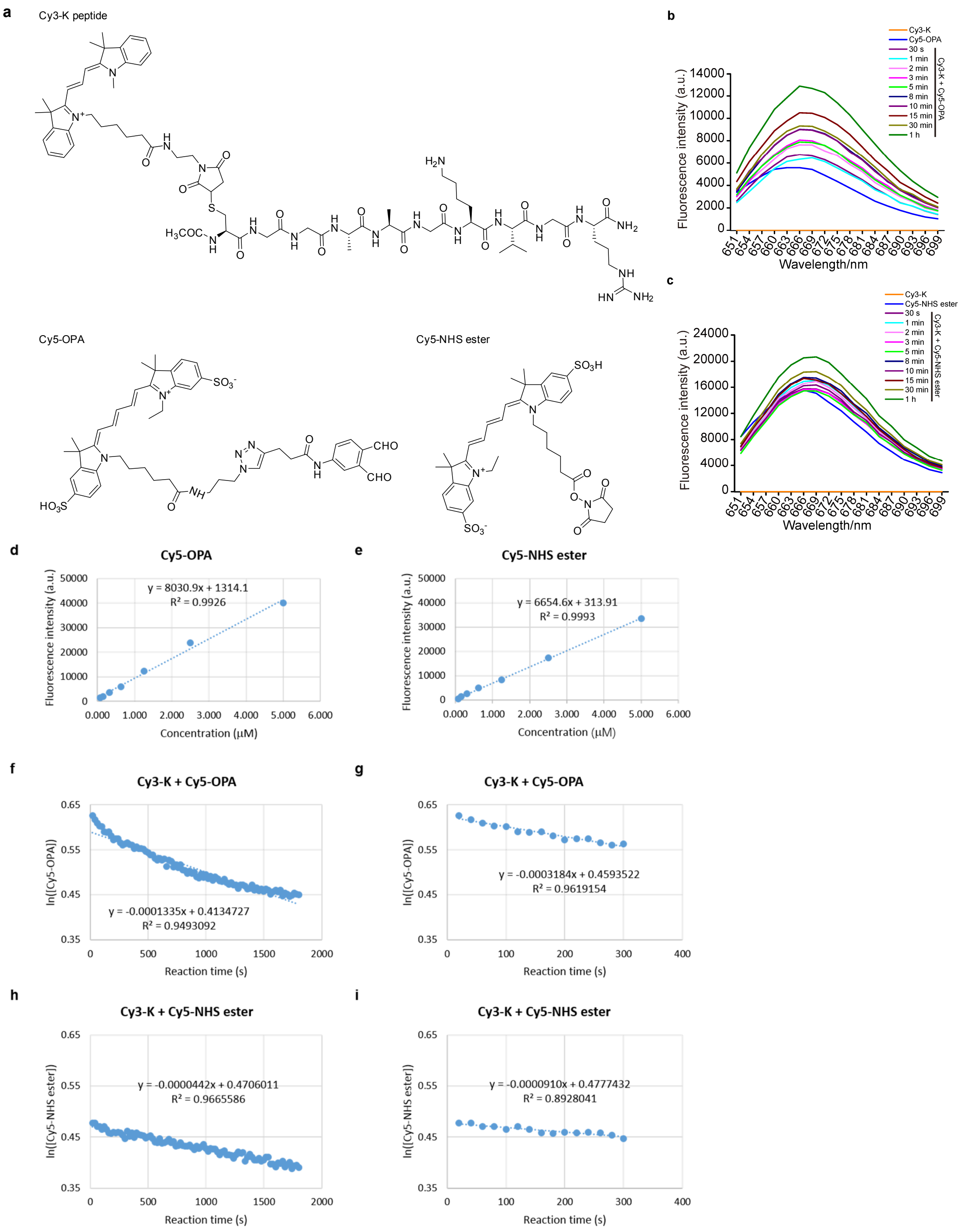

Supplementary Figure 3. FRET-based system comparison of the reaction rate of Cy5-OPA and Cy5-NHS ester with Cy3-K peptide.

(a) The chemical structures of Cy3-K peptide, Cy5-OPA, and Cy5-NHS ester. (b) Fluorescence spectra of Cy3-K, Cy5-OPA, and Cy3-K (12.5 μM) mixed with Cy5-OPA (25 μM) for different amounts of time, subtracting the fluorescence intensity of Cy3-K. In the y-axis, a. u. stands for arbitrary units. (c) As in b, but Cy5-OPA was replaced with Cy5-NHS ester. (d) Standard curve of fluorescence intensity versus Cy5-OPA concentration (excitation/emission = 649/666 nm). (e) Standard curve of fluorescence intensity versus Cy5-NHS ester concentration (excitation/emission = 649/666 nm). (f) The pseudo-first order reaction rate constant of Cy5-OPA, determined by linear regression analysis of ln([Cy5-OPA]) versus reaction time (1800 s). (g) As in f, but the reaction time was 300 s. (h) The pseudo-first order reaction rate constant of Cy5-NHS ester, determined by linear regression analysis of ln([Cy5-NHS ester]) versus reaction time (1800 s). (i) As in h, but the reaction time was 300 s. Source data for b-i are provided as a Source Data file.

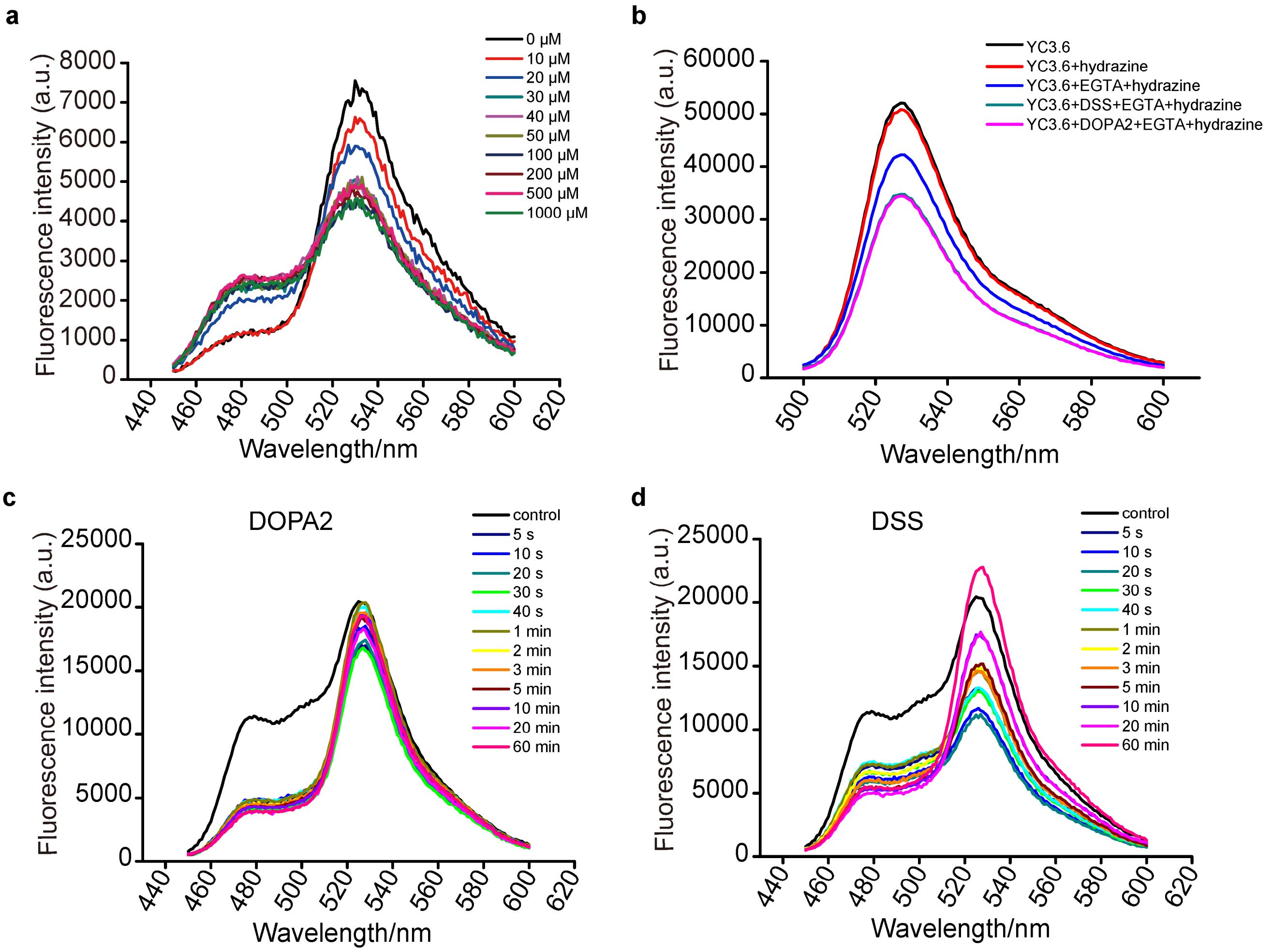

Supplementary Figure 4. Comparing the rates of cross-linking of DOPA2 and DSS based on an EGTA-resistant FRET signal of Yellow Cameleon 3.6 (YC3.6).

(a) Fluorescence spectra of YC3.6 in different concentrations of EGTA (excitation = 420 nm). (b) The YFP fluorescence of YC3.6 was verified in the presence of hydrazine, EGTA, DSS, and DOPA2. (excitation = 480 nm). (c) Fluorescence spectra of YC3.6 cross-linked by DOPA2 for different amount of time (excitation = 420 nm). (d) Fluorescence spectra of YC3.6 cross-linked by DSS for different amount of time (excitation = 420 nm). In the y-axis, a. u. stands for arbitrary units. Source data for a-d are provided as a Source Data file.

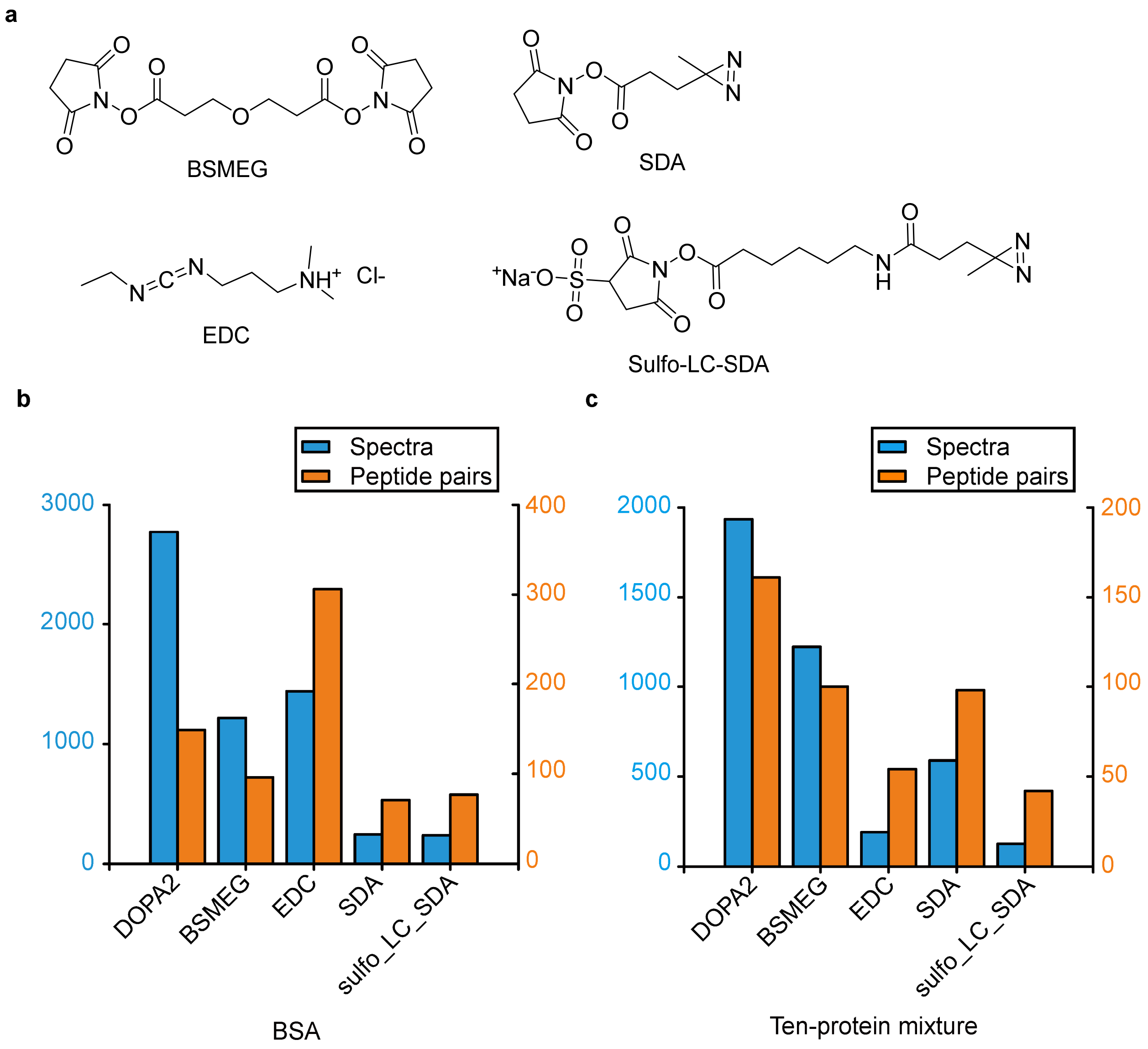

Supplementary Figure 5. A broader comparison between DOPA2 and other cross-linkers.

(a) The chemical structures of BSMEG, EDC, SDA, and Sulfo-LC-SDA. (b) Cross-links identified from BSA using DOPA2, BSMEG, EDC, SDA, and Sulfo-LC-SDA. The numbers of cross-linked spectra are plotted with blue columns, and the number of cross-linked peptide pairs with orange. (c) As in b, but on the ten-protein mixture. Identified cross-links were filtered by requiring FDR < 0.01 at the spectral level. Source data for b and c are provided as a Source Data file.

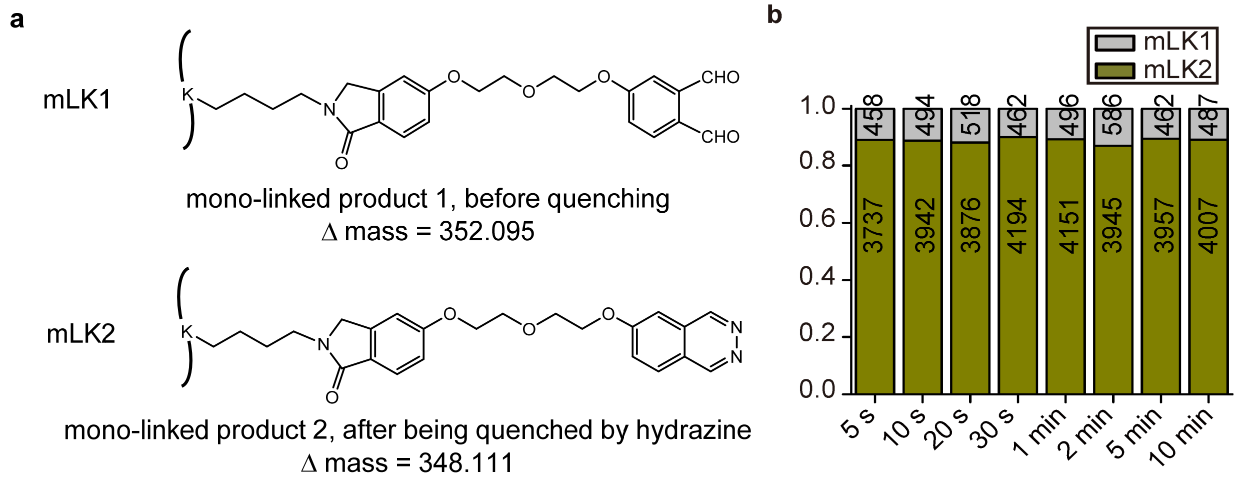

Supplementary Figure 6. DOPA2 cross-linking of BSA was quenched by hydrazine within five seconds.

Ten seconds after DOPA2 (0.17 mM, final conc.) was added to a 1mg/mL BSA solution, hydrazine (20 mM , final conc.) was added to the mixture and let incubate for 5 s, 10 s, … and up to 10 min. Then, six volumes of cool acetone was added to precipitate BSA. After the hydrazine-containing supernatant was quickly removed, the pellet was digested with trypsin and analyzed by LCMS.

(a) Illustration of the structures of mono-linked peptides before (mLK1) and after (mLK2) quenching by hydrazine. (b) Percentage of spectral counts of mLK1 and that of mLK2 after the DOPA2-BSA reaction was quenched by hydrazine for the indicated amount of time. The number of spectra identified is shown on the bar. The data was filtered by requiring FDR < 1% at the spectra level. Source data for b are provided as a Source Data file.

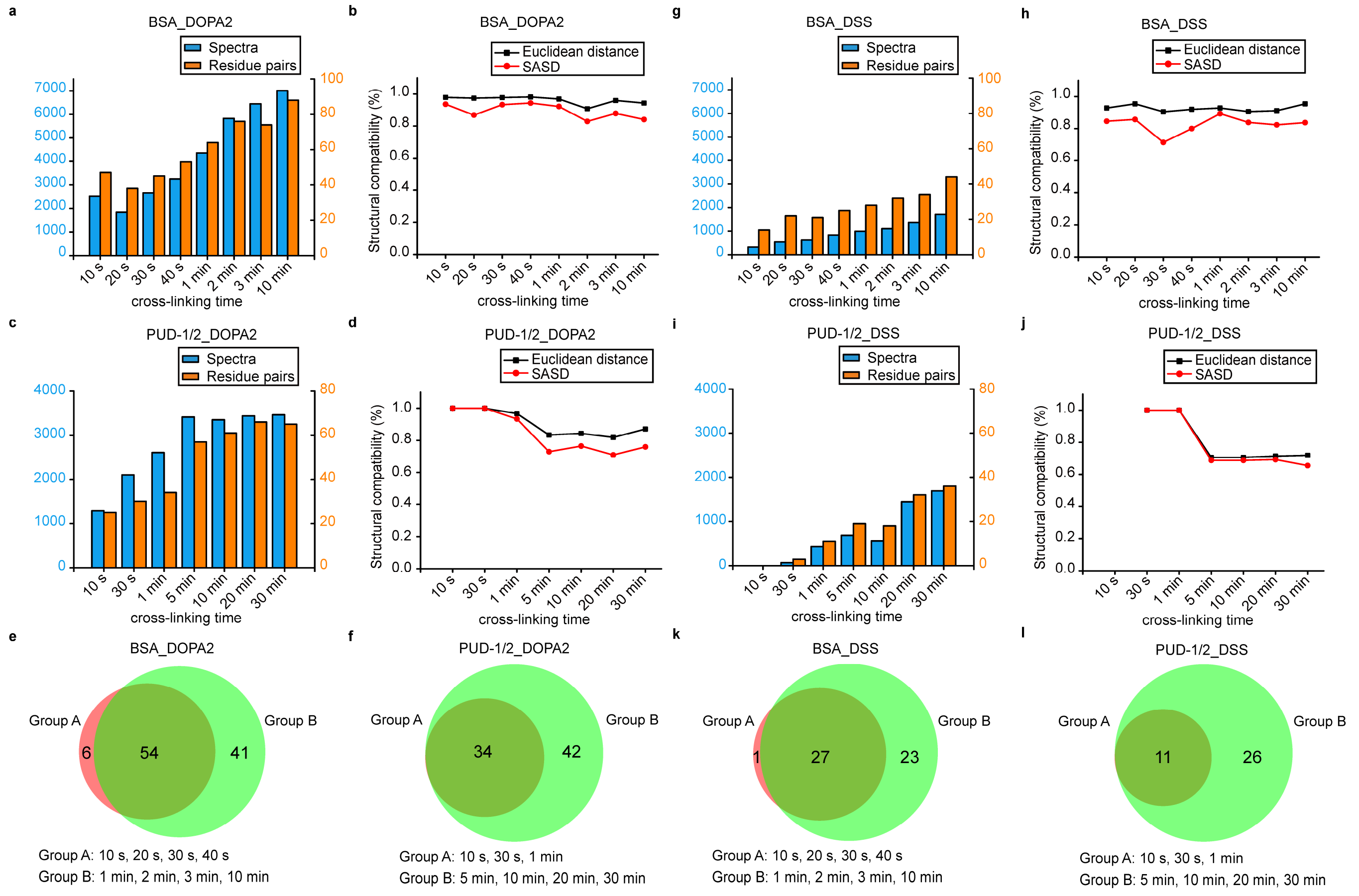

Supplementary Figure 7. Comparing DOPA2 or DSS cross-linking reactions of different durations.

(a) Cross-links identified in BSA after cross-linking by DOPA2 for the indicated amount of time. The number of cross-link spectra is represented by blue columns, and the number of cross-linked residue pairs by orange columns. (b) The fraction of residue pairs that are consistent with the structure of BSA (PDB code: 3V03), as calculated by the use of the Euclidean distance or the solvent accessible surface distance (SASD). (c and d) As in a-b, but for PUD-1/2 complex (PDB code: 4JDE). (e) Venn diagram showing the overlap of DOPA2 cross-links identified in BSA in short reactions and those in longer reactions. The cross-links identified from 10-, 20-, 30-, and 40-s reactions were combined into Group A and the cross-links identified from 1-, 2-, 3-, and 10-min reactions were combined into Group B. (f) As in (e), but for PUD-1/2 complex. (g-l) As in a-f, but cross-linked by DSS. Cross-links were filtered by requiring FDR < 0.01 at the spectra level, E-value < 1×10^{-3}. Source data for a-l are provided as a Source Data file.

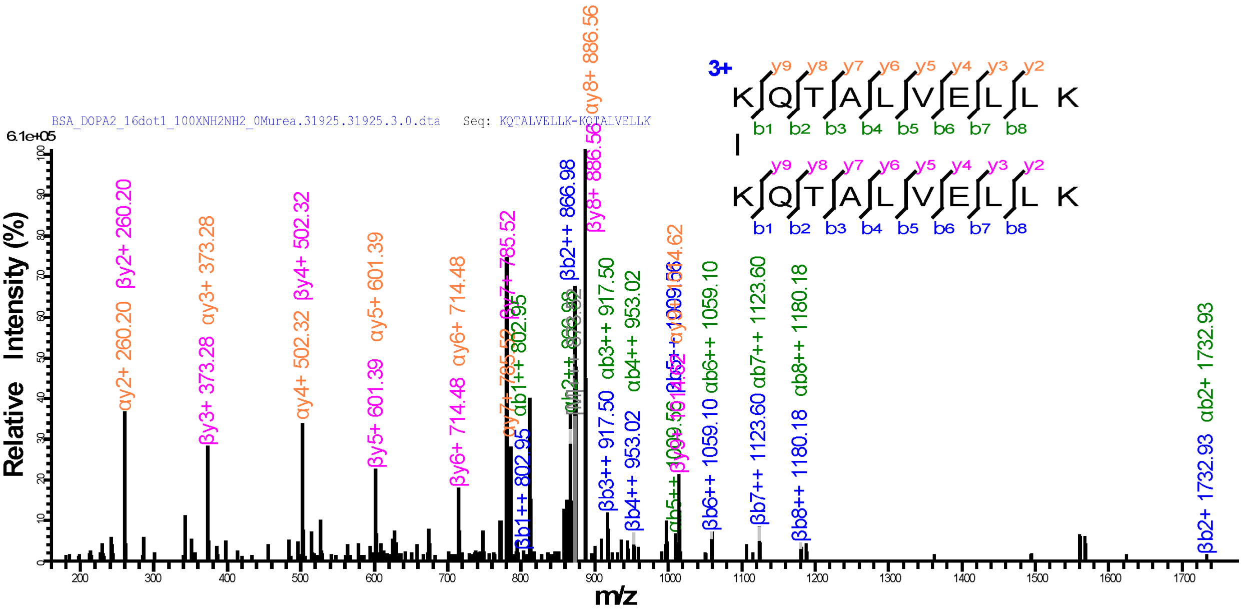

Supplementary Figure 8. Annotated MS/MS spectrum of the DOPA2-linked inter-molecular peptide pair from BSA.

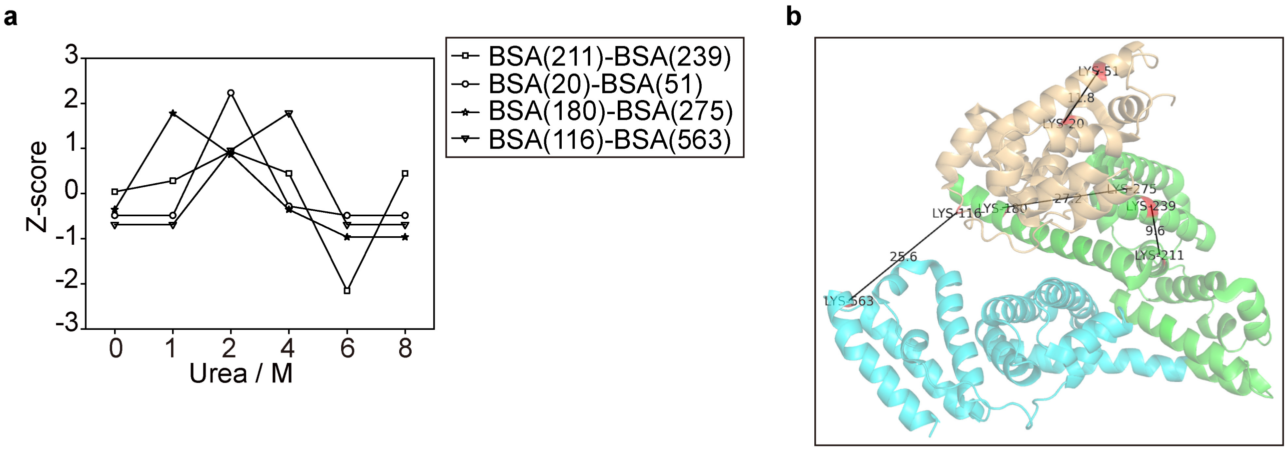

Supplementary Figure 9. The four unclassified cross-links of BSA by DOPA2 cross-linking in urea.

(a) Z-scores reporting the normalized spectral counts of four unclassified cross-links as a function of urea concentration. (b) The cross-links are indicated on the crystal structure of BSA (PDB code: 3V03). Source data for a are provided as a Source Data file.

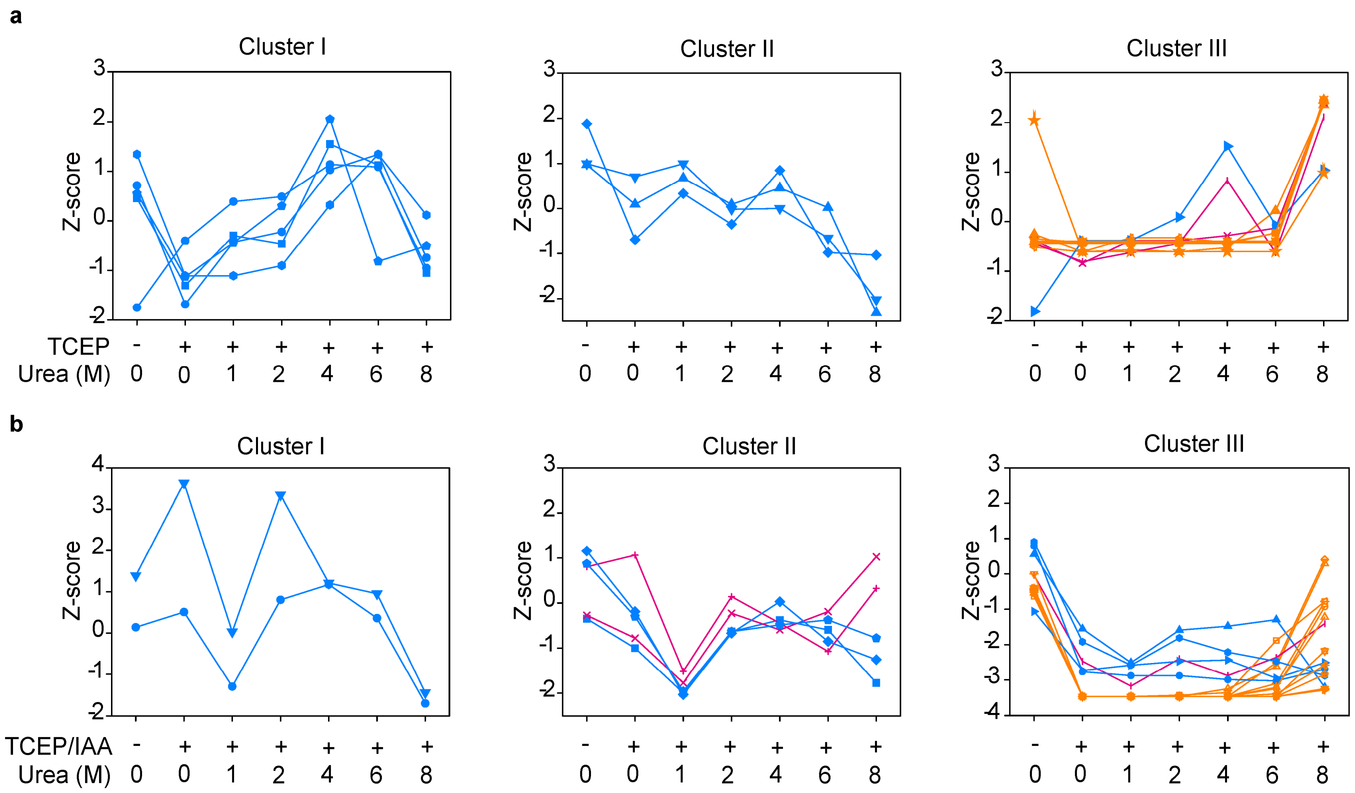

Supplementary Figure 10. Analysis of the unfolding process of RNase A in urea using DOPA2 under reduction conditions.

(a) The changes of spectral counts for each identified cross-linked residue pair in different concentrations of urea. RNase A samples were reduced by TCEP ahead of denaturation in urea buffer. The residue pairs identified were classified into three clusters by K-means (Cluster I, Cluster II, and Cluster III). The coloring of cross-links is consistent with Cluster A, Cluster B, and Cluster C in Figure 7d. (b) Similar to a, but RNase A samples were reduced by TCEP and alkylated by IAA ahead of denaturation in urea buffer. Cross-linking residue pairs were filtered by requiring FDR < 0.01 at the spectra level, E-value $< 1\times10^{-8}$, and spectral counts > 3. Source data for a-b are provided as a Source Data file.

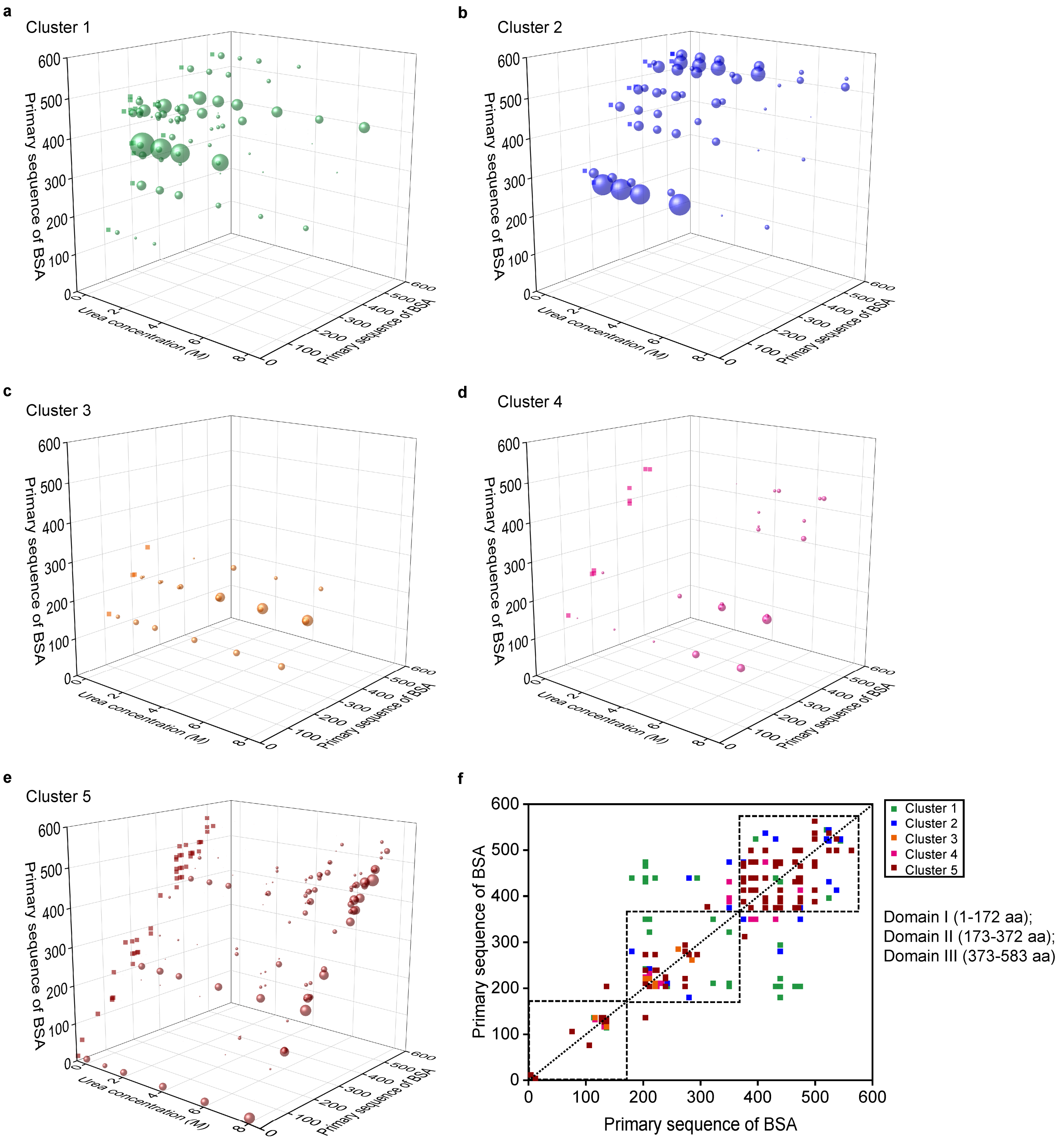

Supplementary Figure 11. Scatter plot of DOPA2 cross-links on the primary sequence of BSA.

(a-e) 3D scatter plot of the cross-links in Cluster 1-5 on the primary sequence of BSA with size representing the spectral counts (ball size = spectral counts$^{0.5}$ *2). (f) 2D scatter plot of DOPA2 cross-links on the primary sequence of BSA with colors representing the above five clusters. Source data for a-f are provided as a Source Data file.

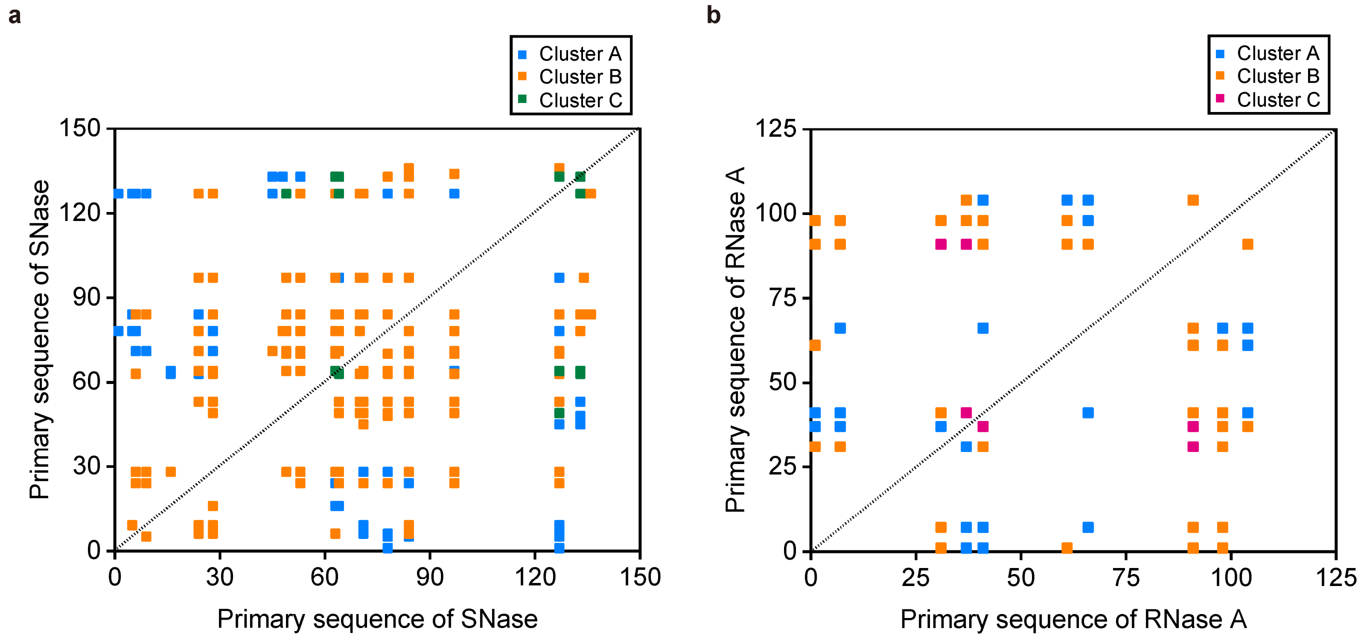

Supplementary Figure 12. Scatter plots of DOPA2 cross-links on the primary sequences of SNase and RNase A.

(a) 2D scatter plot of DOPA2 cross-links on the primary sequence of SNase with colors representing three clusters. (b) 2D scatter plot of DOPA2 cross-links on the primary sequence of RNase A with colors representing three clusters. Source data for a-b are provided as a Source Data file.

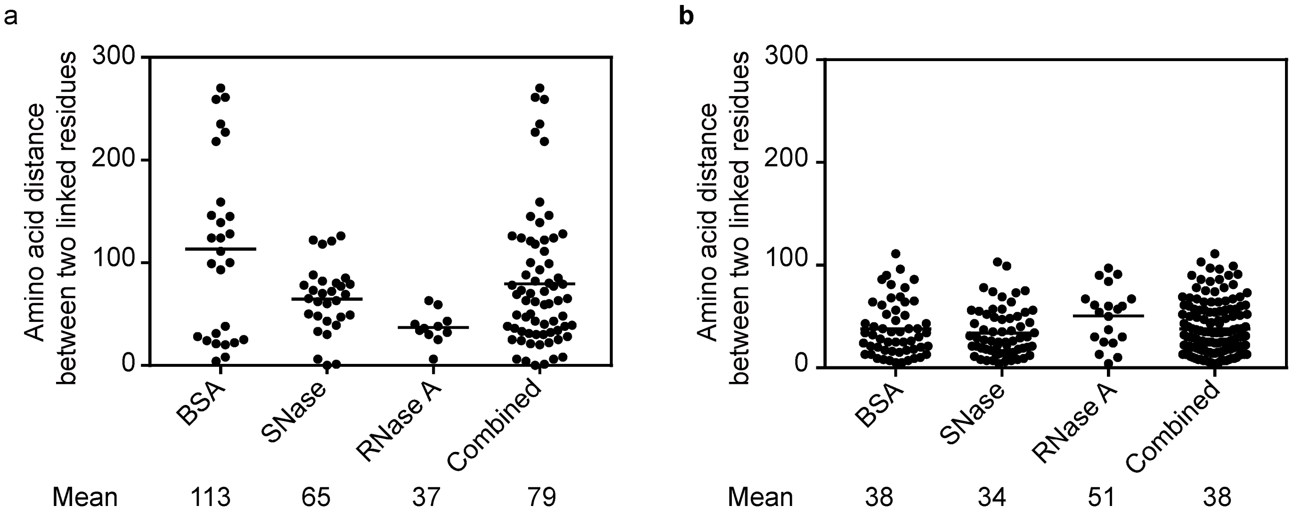

Supplementary Figure 13. Distribution of the amino acid distances between two linked residues of the native versus the non-native clusters of DOPA2 cross-links.

(a) Distribution of the amino acid distances between two linked residues in native clusters. n = 27, 30, 11, and 68 residue pairs for BSA, SNase, RNase A, and Combined, respectively, examined over two independent experiments. (b) Distribution of the amino acid distances between two linked residues in non-native clusters. n = 55, 64, 20, and 139 residue pairs for BSA, SNase, RNase A, and Combined, respectively, examined over two independent experiments. Source data for a-b are provided as a Source Data file.

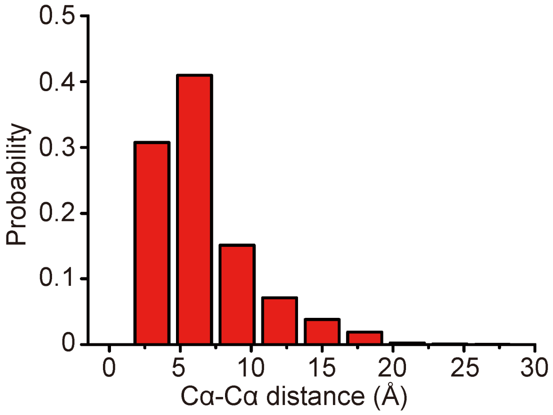

Supplementary Figure 14. The Cα-Cα distance distribution of DOPA2 obtained by MD simulations. Source data are provided as a Source Data file.

Supplementary Table 1. Peptides tested for OPA selectivity.

Peptide	Sequence	$[M+H]^+$
TR-8	TPDVNKDR	944.4796
VR-7	VWDLVKR	915.5410
KR-7	KMRPEVR	915.5193
GR-11	(N,N-dimethyl-Gly)-VAAAKAAAAR	984.5949
VR-6	VKTELR	745.4566
NR-9	NSKIFSPFR	1095.5945
VK-9	VGGSTIKSK	876.5149
YK-14	YFAYISKLDSASVK	1591.8366
HR-9	HGPVCAKYR	1030.5251
FR-9	FVKQQWNLR	1218.6742

Supplementary Table 2. Products of ten synthesized peptides and OPA.

For each product, the mass-shift from the intact peptide is indicated, and the relative abundance is calculated based on the chromatographic peak areas from the corresponding. (X = N, S, or O; Xaa = Lys, Cys, Tyr, or the peptide N-terminus)

Chemical structure	Mass-shift from intact peptide	Attachment site	TR-8	VR-7	KR-7	GR-11	VR-6	NR-9	VK-9	YK-14	HR-9	FR-9
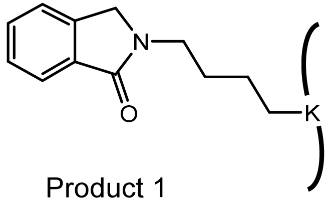 Product 1	+116.0262 Da	K	0.58	0.48	0.43	1.00	0.55	-	0.21	-	0.12	0.48
		N-terminus	0.14	0.31	0.02	-	0.35	0.61	0.13	0.12	-	0.25
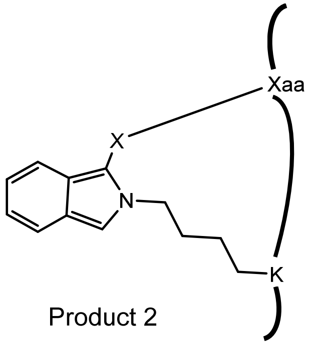 Product 2	+98.0156 Da	K and N-terminus	0.28	0.21	0.43	-	0.08	0.35	0.30	0.45	-	0.27
		K and K	-	-	-	-	-	-	0.08	-	-	-
		Y and N-terminus	-	-	-	-	-	-	-	0.43	-	-
		C and K/N-terminus	-	-	-	-	-	-	-	-	0.88	-
Unknown	+2.9421 Da		-	-	0.04	-	-	-	-	-	-	-
	+232.0535 Da		-	-	0.06	-	-	-	-	-	-	-
	-12.0693 Da		-	-	0.02	-	-	-	-	-	-	
	+114.9962 Da		-	-	-	-	0.02	-	-	-	-	-
	+114.0140 Da		-	-	-	-	-	0.05	-	-	-	-
	+98.0184 Da		-	-	-	-	-	-	0.27	-	-	-

Supplementary Table 3. The composition of the ten-protein mixture and the PDB code of the proteins in Figure 3d.

(a) The composition of a ten-protein mixture and the molecular weight for each protein. (b) The PDB code of the proteins in Figure 3d. Note: due to the lack of the corresponding PDB code, myosin is not included when calculating the structural compatibility rate.

a

#	Protein Name	MW (kDa)
1	myosin	242
2	lactoferrin	78
3	BSA	69
4	catalase	60
5	β-amylase	56
6	aldolase	39
7	carbonic anhydrase 2	29
8	GST	28
9	PUD-1/PUD-2 heterodimer	17/17
10	lysozyme	16

b

Protein name	PDB code
aldolase	1ZAH
BSA	3V03
catalase	5GKN
GST	1Y6E
lysozyme	1LYZ

Supplementary Table 4. The intensity of different products in peptide samples cross-linked by DOPA2 or DSS at low pH and in the presence of denaturants.

(a) The chromatographic base peak intensity of different products in peptide samples cross-linked by DOPA2 in low pH buffer and in physiological buffer. “Intact peptide” refers to free peptides without cross-linking. “X-link” refers to the situation wherein two peptides are linked with one molecule of DOPA2. “Mono-link” refers to a peptide that has been modified but is not cross-linked by a cross-linker. (b) The chromatographic base peak intensity of different products in peptide samples cross-linked by DSS in low pH buffer and in physiological buffer. (c) The chromatographic base peak intensity of different products in peptide samples cross-linked by DOPA2 in 6 M GdnHCl buffer or HEPES buffer.

a

	DOPA2							
	HEPES Buffer, pH7.4				Citric Acid - Na_2HPO_4 Buffer, pH3.0			
Peptide sequence	Intact peptide (A)	X-link (B)	Mono-link (C)	(B+C)/(A+B+C)	Intact peptide (A)	X-link (B)	Mono-link (C)	(B+C)/(A+B+C)
TR-8	5.34E+07	2.36E+06	7.19E+07	0.58156	1.07E+08	1.56E+06	5.45E+07	0.34478
VR-7	8.33E+09	2.63E+06	2.24E+08	0.02647	3.72E+09	9.15E+06	9.88E+08	0.21130
KR-7	2.36E+08	7.38E+08	7.38E+08	0.86227	1.91E+06	0.00E+00	3.57E+08	0.99469
GR-11	8.40E+09	1.52E+09	1.52E+09	0.26542	1.37E+10	4.02E+06	3.68E+09	0.21221

b

	DSS							
	HEPES Buffer, pH7.4				Citric Acid - Na_2HPO_4 Buffer, pH3.0			
Peptide sequence	Intact peptide (A)	X-link (B)	Mono-link (C)	(B+C)/(A+B+C)	Intact peptide (A)	X-link (B)	Mono-link (C)	(B+C)/(A+B+C)
TR-8	1.12E+08	0	6.00E+07	0.34863	8.77E+08	0	3.33E+04	0.00004
VR-7	6.16E+09	0	5.32E+08	0.07951	1.11E+10	0	3.97E+07	0.00357
KR-7	1.03E+08	0	5.07E+08	0.83055	9.69E+07	0	2.23E+06	0.02245
GR-11	9.89E+09	0	1.53E+06	0.00015	1.53E+10	0	1.89E+06	0.00012

c

	DOPA2							
	HEPES Buffer, pH7.4				6 M GdnHCl			
Peptide sequence	Intact peptide (A)	X-link (B)	Mono-link (C)	(B+C)/(A+B+C)	Intact peptide (A)	X-link (B)	Mono-link (C)	(B+C)/(A+B+C)
VR-7	1.71E+10	8.27E+08	6.37E+09	0.29607	2.37E+10	2.56E+09	2.77E+10	0.56072
GR-11	3.01E+08	1.55E+10	1.00E+10	0.98834	7.47E+08	3.01E+08	1.51E+10	0.95381

^{1}H NMR and ^{13}C NMR spectra

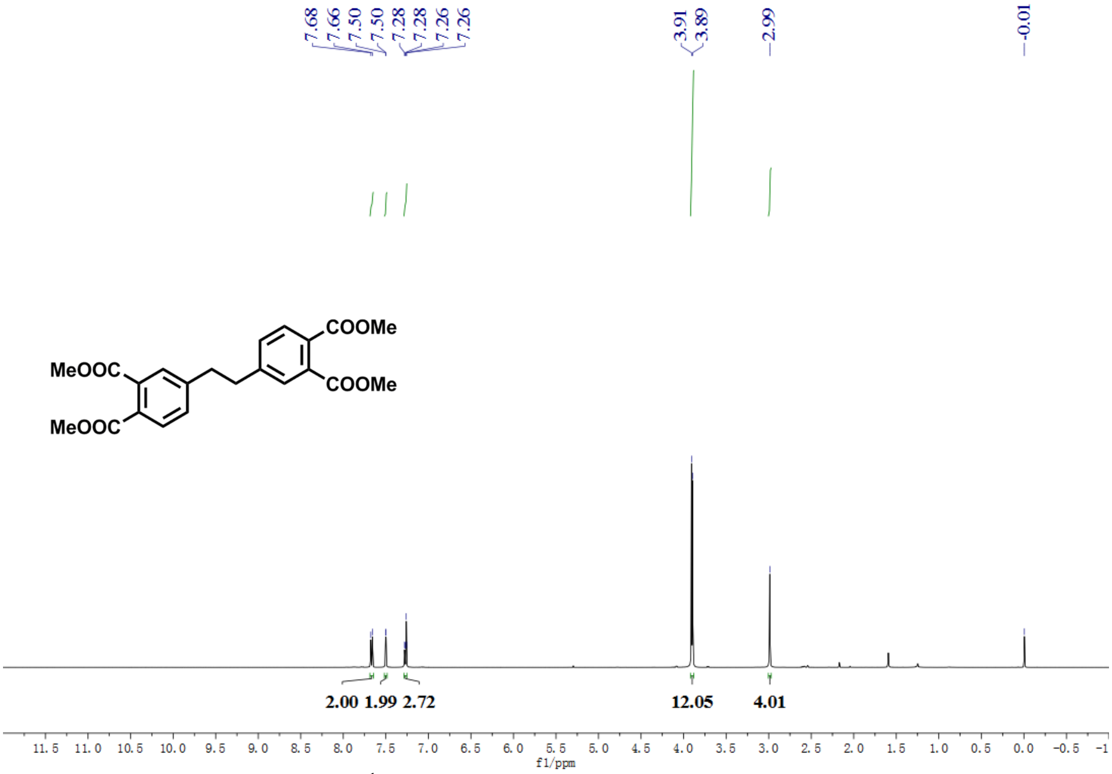

Supplementary Figure 15. ^{1}H NMR of compound 2 (400 MHz, $CDCl_3$)

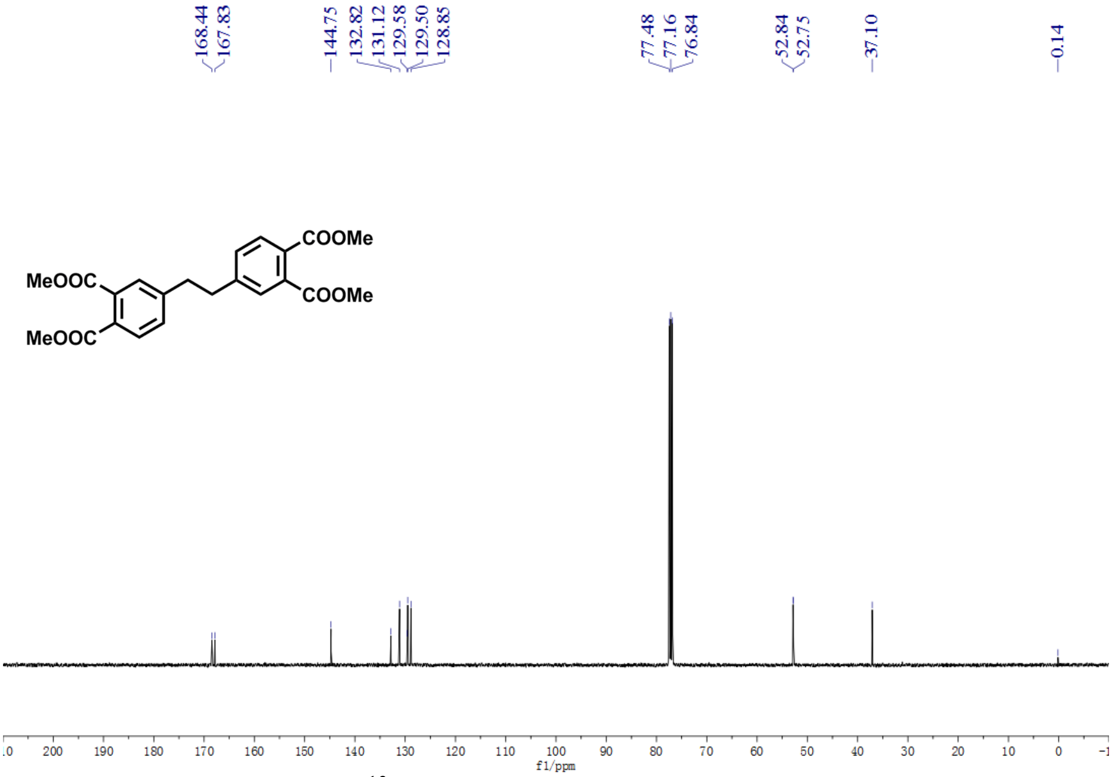

Supplementary Figure 16. ^{13}C NMR of compound 2 (100 MHz, $CDCl_3$)

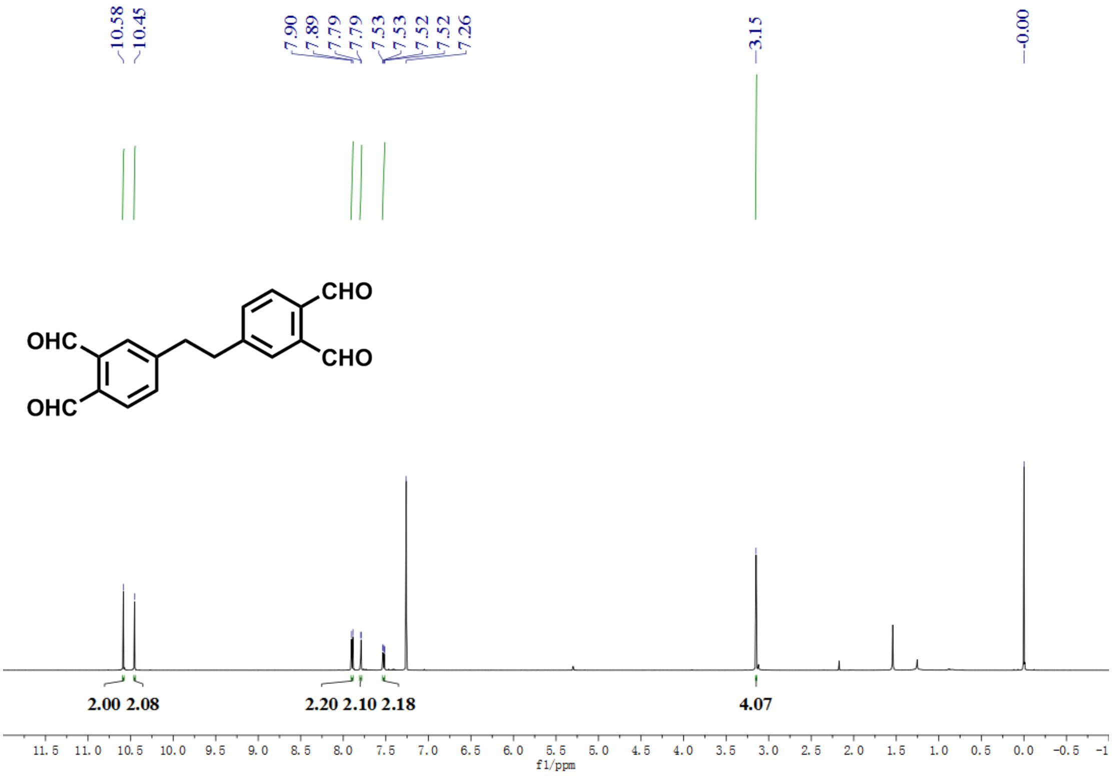

Supplementary Figure 17. ^{1}H NMR of compound 4 (500 MHz, $CDCl_3$)

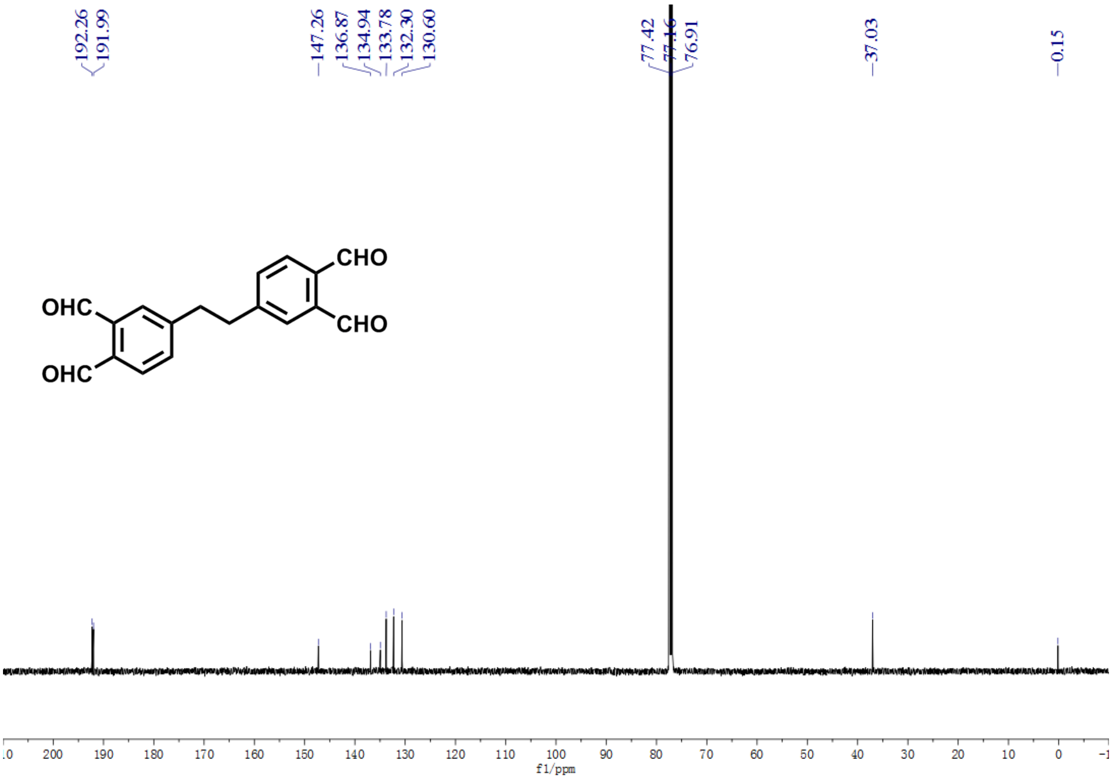

Supplementary Figure 18. ^{13}C NMR of compound 4 (125 MHz, $CDCl_3$)

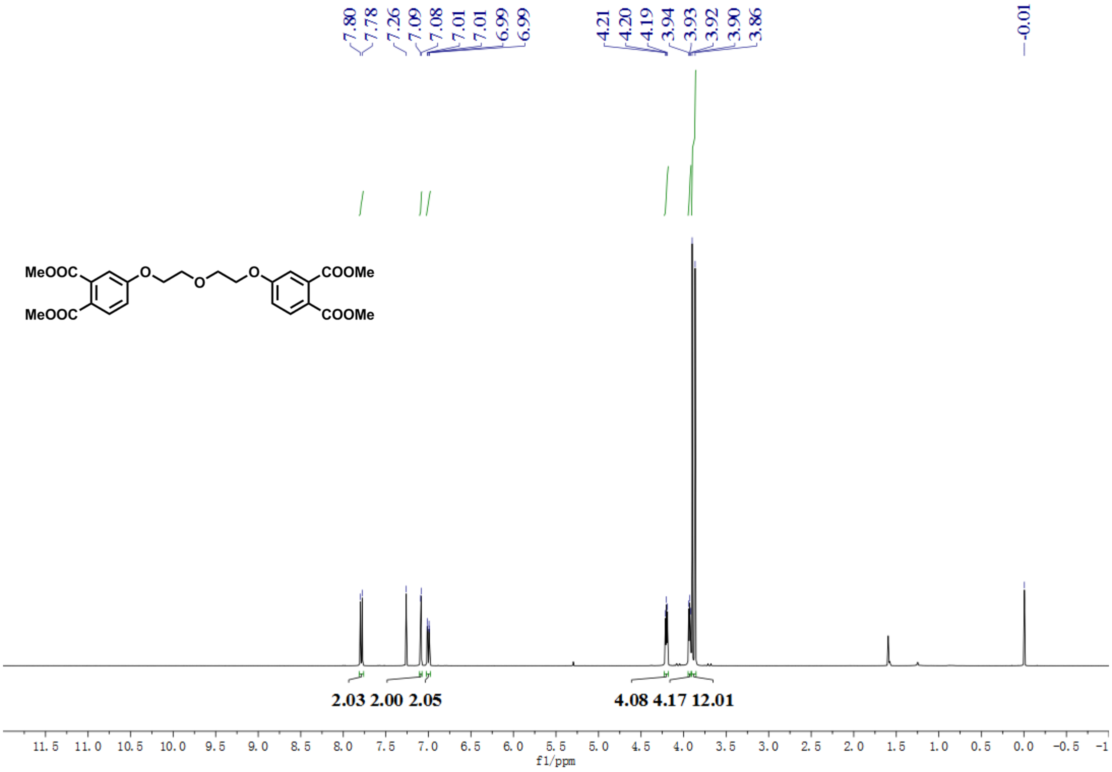

Supplementary Figure 19. ^{1}H NMR of compound 6 (400 MHz, $CDCl_3$)

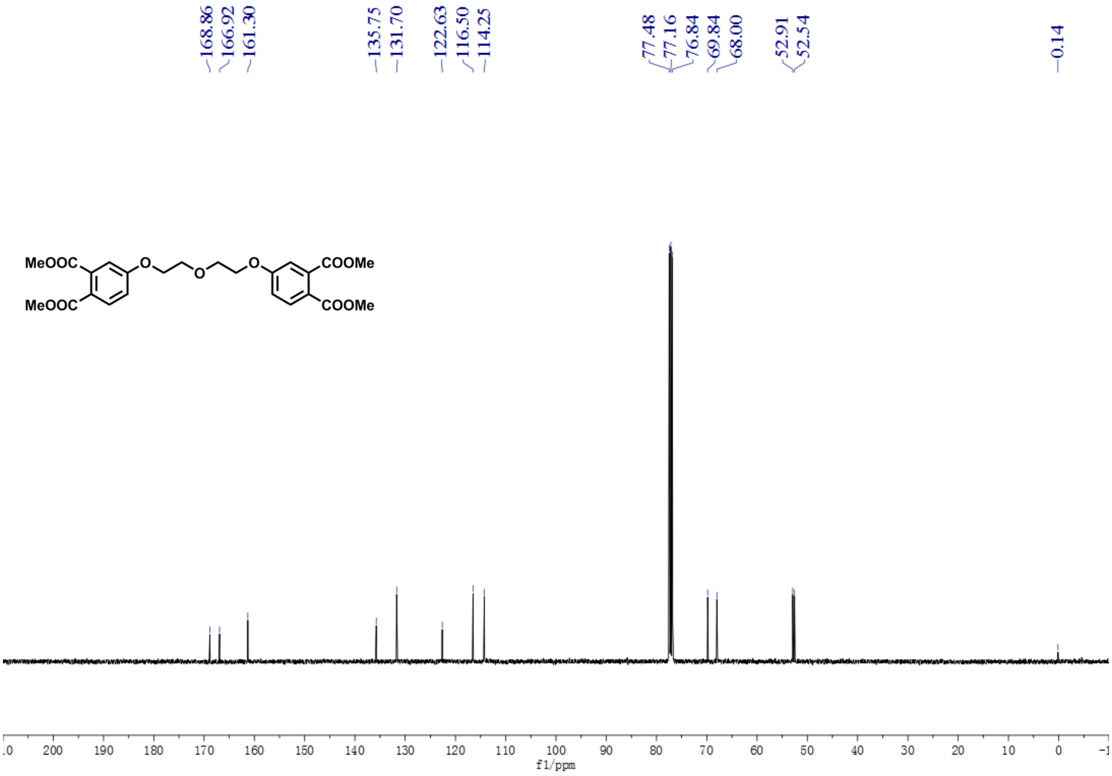

Supplementary Figure 20. ^{13}C NMR of compound 6 (100 MHz, $CDCl_3$)

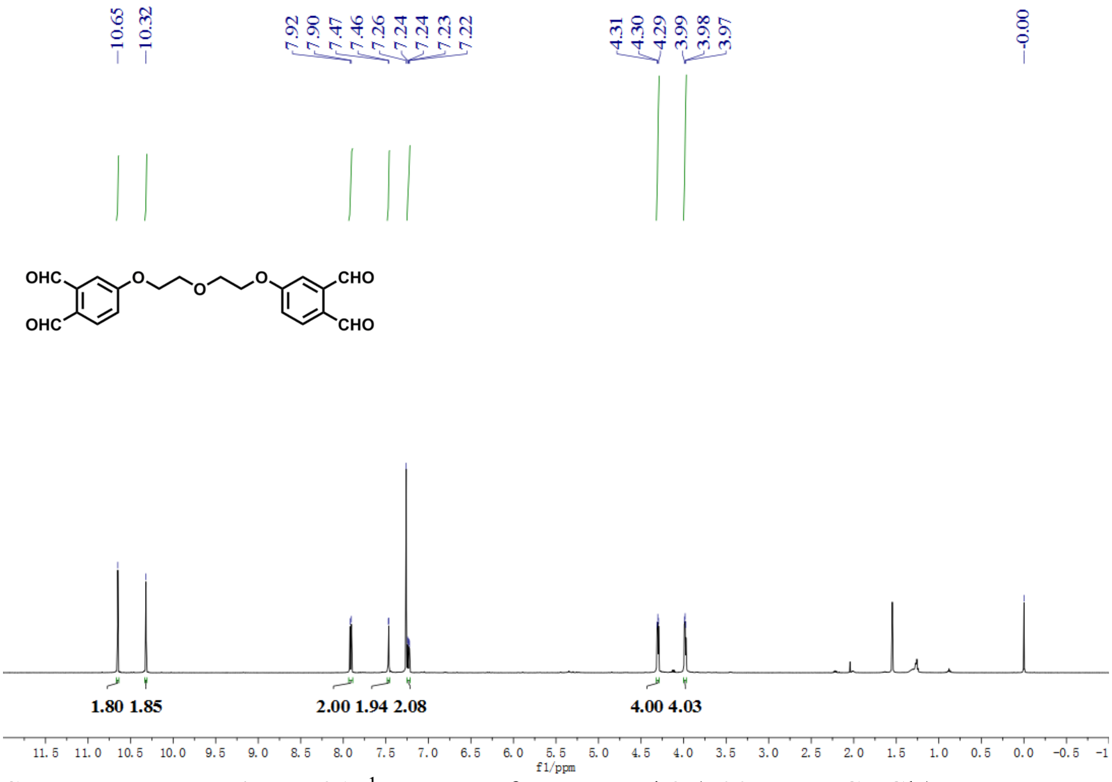

Supplementary Figure 21. ^{1}H NMR of compound 8 (500 MHz, $CDCl_3$)

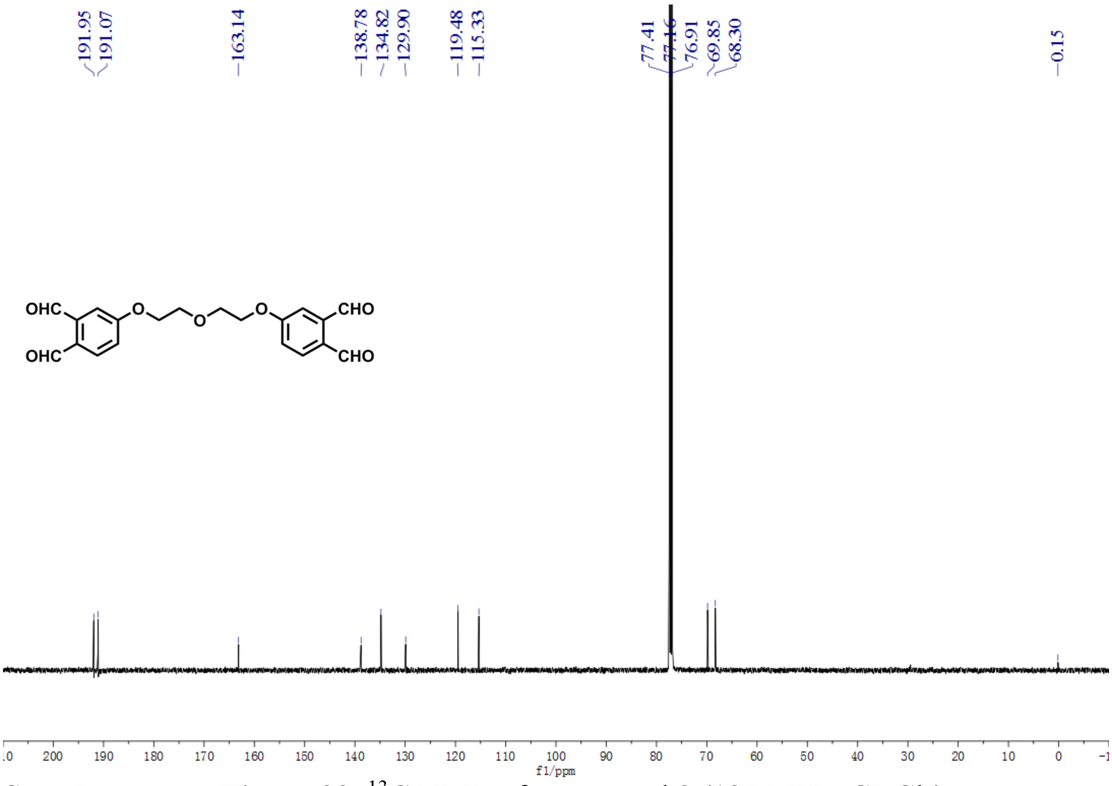

Supplementary Figure 22. ^{13}C NMR of compound 8 (125 MHz, $CDCl_3$)

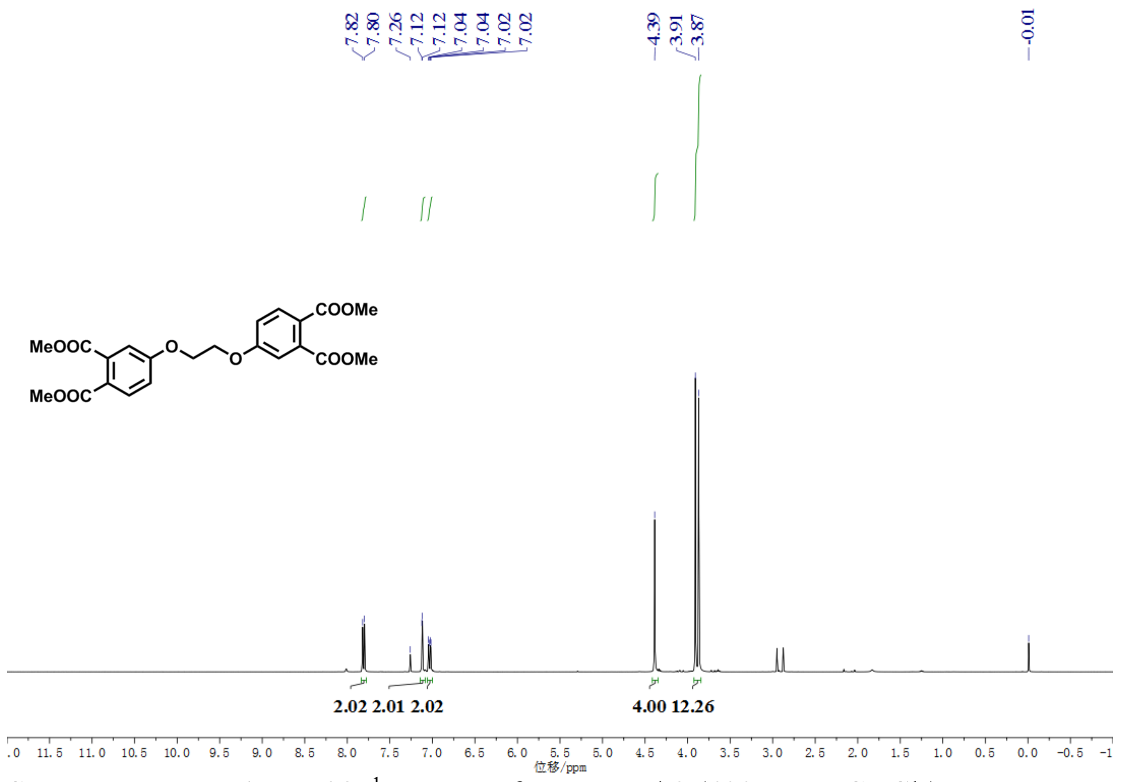

Supplementary Figure 23. ^{1}H NMR of compound 9 (400 MHz, $CDCl_3$)

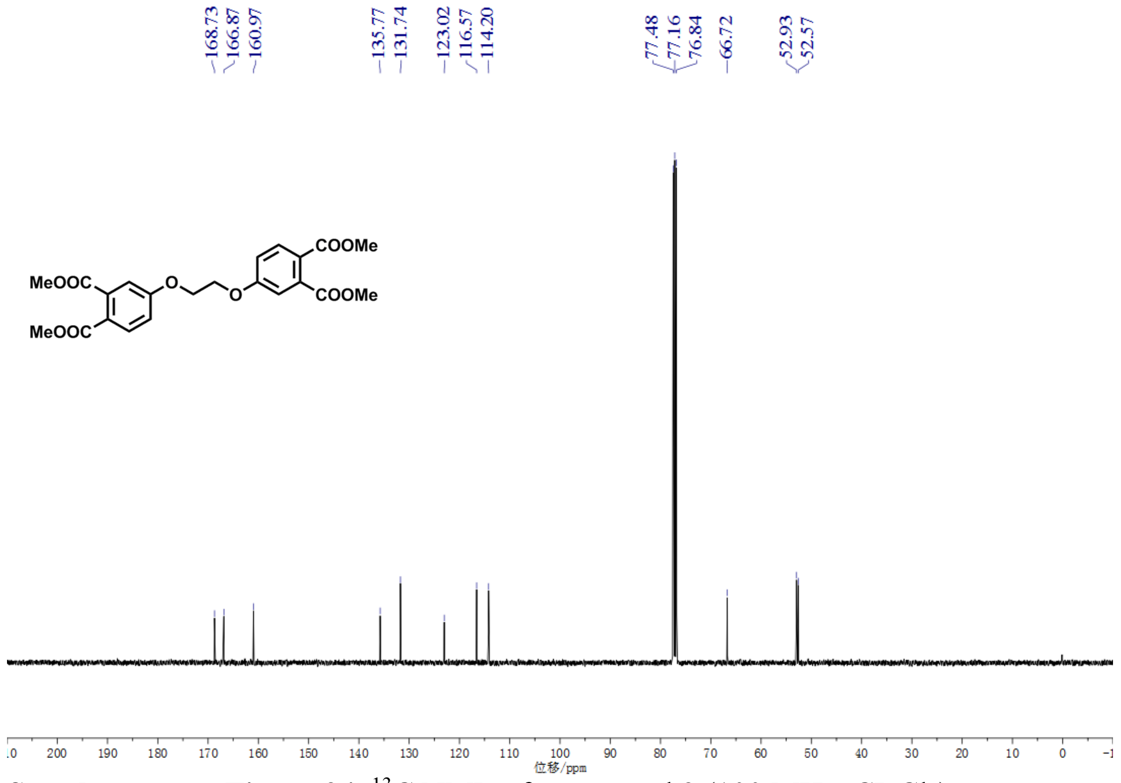

Supplementary Figure 24. ^{13}C NMR of compound 9 (100 MHz, $CDCl_3$)

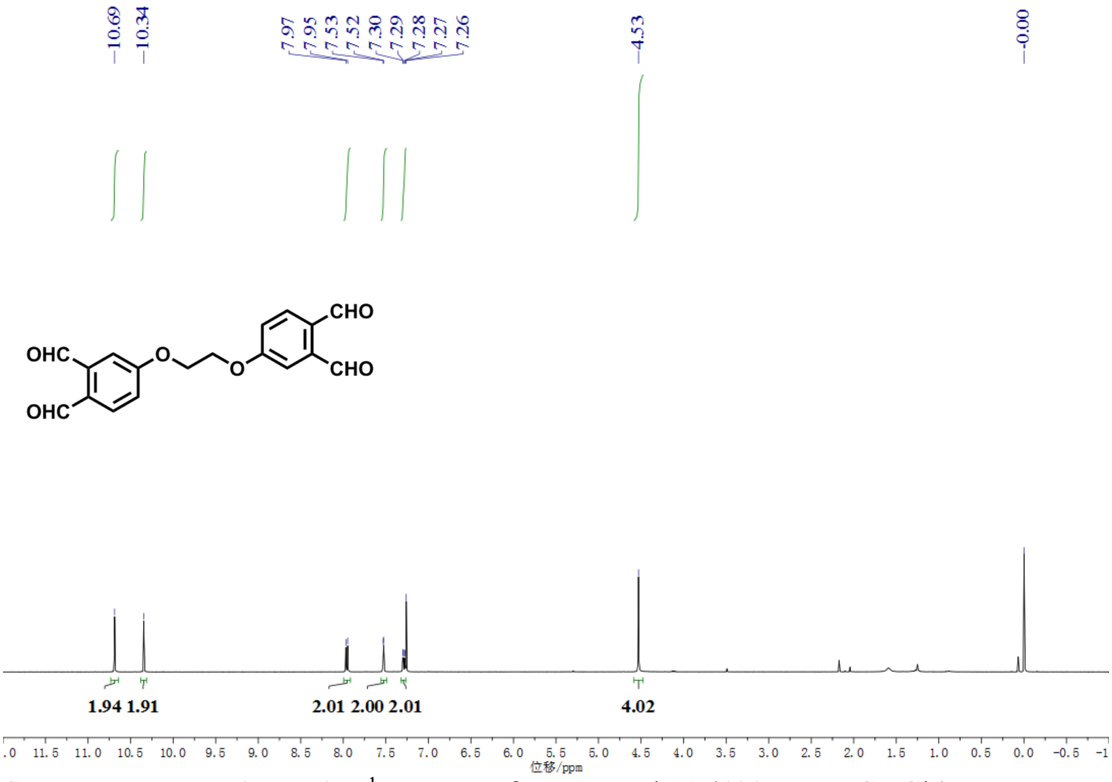

Supplementary Figure 25. ^{1}H NMR of compound 11 (400 MHz, $CDCl_3$)

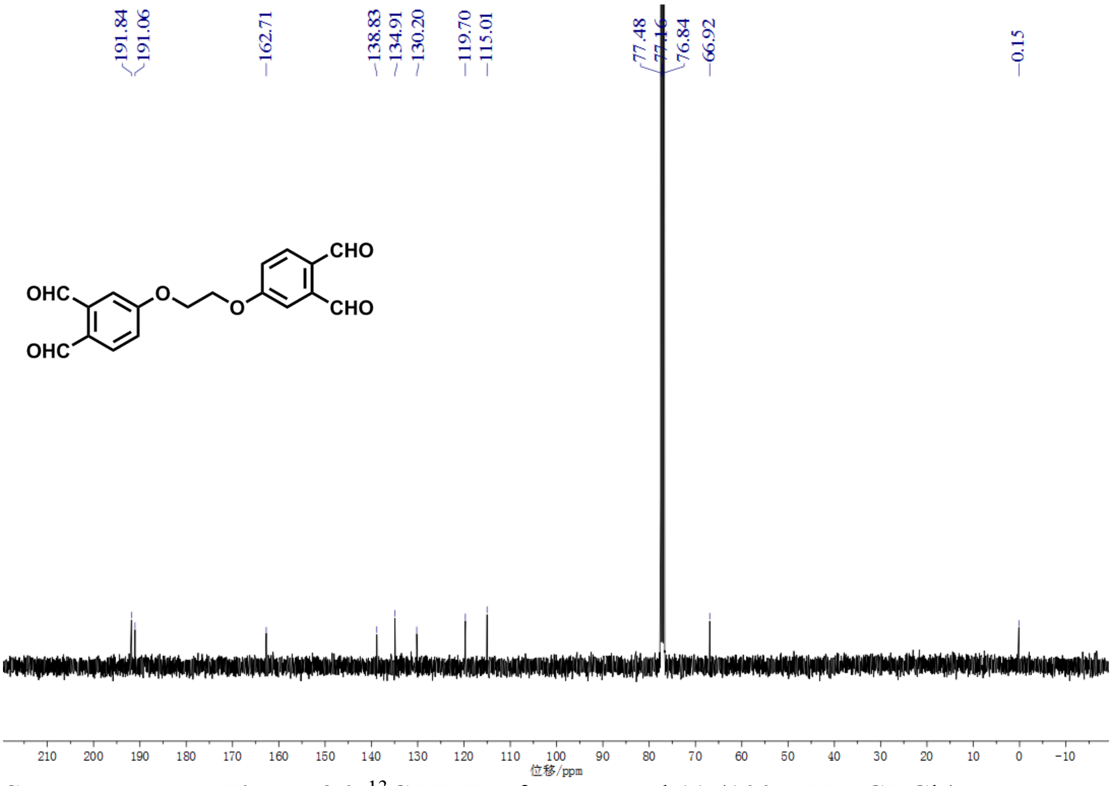

Supplementary Figure 26. ^{13}C NMR of compound 11 (100 MHz, $CDCl_3$)

Supplementary Methods

Synthetic procedures and analytical data

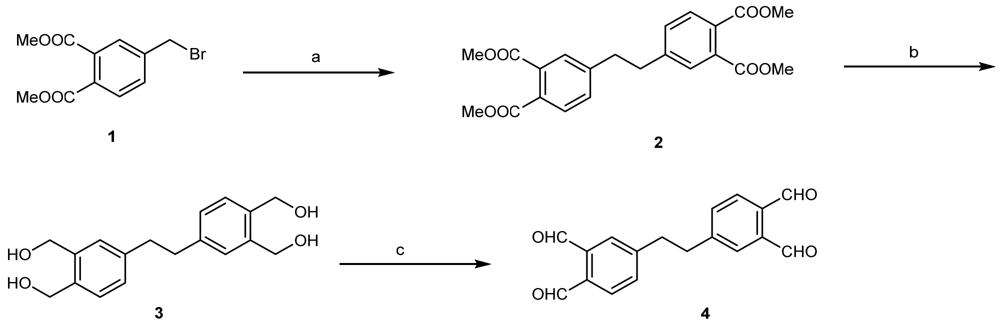

Reagents and conditions: (a) Me_2Zn, $RhCl(PPh_3)_3$, THF, 24 h, 76%; (b) DIBAL-H, THF, 12 h; (c) Dess-Martin periodinane, DCM, 12 h, 98% for two steps.

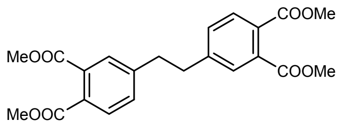

2

Compound **2**: To a solution of RhCl $(PPh_3)_3$ (18.5 mg, 0.0200 mmol) and compound **1** (0.287 g, 1.00 mmol) in anhydrous and thoroughly degassed THF (5.00 mL) was added Me_2Zn (1.00 mL, 1.00 mmol, 1.0 M in hexanes) at room temperature. The resulting mixture was stirred under argon atmosphere for 24 h. After that, the reaction mixture was quenched with 2 M HCl (5.0 mL), and extracted with EtOAc (20.0 mL×3). The combined organic layers were washed with brine (10.0 mL). After dried over Na_2SO_4, the solution was concentrated *in vacuo* and purified by silica gel flash chromatography (petrol ether/EtOAc, 4:1 - 7:3) to afford the desired compound **2** as a white solid (0.315 g, 76%).

1H NMR (400 MHz, CDCl3): δ 7.67 (d, J = 8.0 Hz, 2H), 7.50 (d, J = 2.0 Hz, 2H), 7.27 (dd, J = 8.0, 2.0 Hz, 2H), 3.90 (d, J = 8.0 Hz, 12H), 2.99 (s, 4H); 13C NMR (100 MHz, CDCl3): δ 168.4, 167.8, 144.7, 132.8, 131.1, 129.6, 129.5, 128.8, 52.8, 52.7, 37.1; HRMS (m/z): calcd. for C22H23O8, 414.1387; found, 415.1387.

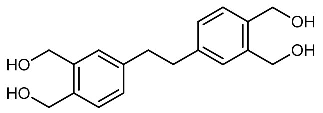

3

Compound **3**: To a solution of compound **2** (0.127 g, 0.310 mmol) in anhydrous THF (9.00 mL) was added DIBAL-H (3.00 mL, 3.00 mmol, 1.0 M in hexanes) at room temperature. The resulting mixture was stirred under argon atmosphere for 12 h. After that, the reaction mixture was quenched with potassium sodium tartrate solution (10.0 mL). Most organic solvents were removed under reduced pressure and the mixture was extracted with *i*-PrOH:$CHCl_3$ = 1:3 (40.0 mL×3). The combined organic layers were washed with brine (10.0 mL). After dried over Na_2SO_4, the solution was concentrated *in vacuo* to afford the desired compound **3** as a white solid without further purification.

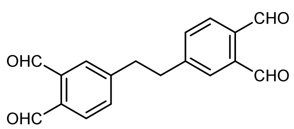

4

Compound **4**: To a solution of compound **3** (90.0 mg, 0.298 mmol) in anhydrous DCM (18.0 mL) was added Dess-Martin periodinane (1.27 g, 3.70 mmol) at room temperature. The resulting mixture was stirred under argon atmosphere for 12 h. After that, the reaction mixture was quenched with Sat. $NaHCO_3$ (10.0 mL) and Sat. $Na_2S_2O_3$ (10.0 mL). The mixture was stirred for 1 h and extracted with DCM (30.0 mL×3). The combined organic layers were washed with brine (10.0 mL). After dried over Na_2SO_4, the solution was concentrated *in vacuo* and purified by silica gel flash chromatography (petrol ether/EtOAc, 4:1 - 2:3 with 5% dichloromethane) to afford the desired compound **4** as a white solid (86.7 mg, 98% for two steps).

1H NMR (500 MHz, CDCl3): δ 10.58 (s, 2H), 10.45 (s, 2H), 7.89 (d, J = 8.0 Hz, 2H), 7.79 (d, J

= 2.0 Hz, 2H), 7.52 (dd, J = 8.0, 2.0 Hz, 2H), 3.15 (s, 4H); 13C NMR (125 MHz, CDCl3): δ 192.3, 192.0, 147.3, 136.9, 134.9, 133.8, 132.3, 130.6, 37.0; HRMS (m/z): calcd. for C18H15O4, 295.0970; found, 295.0965.

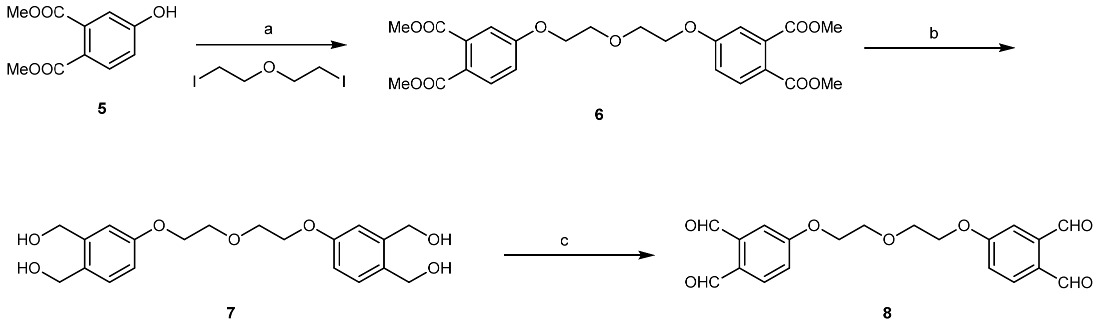

Reagents and conditions: (a) Cs_2CO_3, DMF, 80 °C, 12 h, 77%; (b) DIBAL-H, THF, 50 °C, 2.5 h; (c) Dess-Martin periodinane, DCM, 12 h, 93% for two steps.

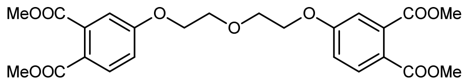

6

Compound **6**: To a solution of Cs_2CO_3 (0.600 g, 1.84 mmol) and compound **5** (0.322 g, 1.53 mmol) in anhydrous DMF (6.00 mL) was added 1,5-diiodo-3-oxopentane (0.200 g, 0.610 mmol) at 80 °C. The resulting mixture was stirred under argon atmosphere for 12h. After that, the reaction mixture was filtered to remove the solid then the filtrate was concentrated *in vacuo* and purified by silica gel flash chromatography (petrol ether/EtOAc, 4:1 - 1:1) to afford the desired compound **6** as a white solid (0.578 g, 77%).

1H NMR (400 MHz, CDCl3): δ 7.79 (d, J = 8.0 Hz, 2H), 7.09 (d, J = 2.0 Hz, 2H), 7.00 (dd, J = 8.0, 2.0 Hz, 2H), 4.20 (m, 4H), 3.93 (m, 4H), 3.88 (d, J = 16.0 Hz, 12H); 13C NMR (100 MHz, CDCl3): δ 168.9, 166.9, 161.3, 135.7, 131.7, 122.6, 116.5, 114.2, 69.8, 68.0, 52.9, 52.5; HRMS (m/z) calcd. for C24H27O11, 491.1553; found, 491.1548.

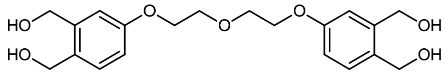

7

Compound **7**: To a solution of compound 6 (73.5 mg, 0.150 mmol) in anhydrous THF (3.50 mL) was added DIBAL-H (1.50 mL, 1.50 mmol, 1.0 M in hexanes) at room temperature. The resulting mixture was stirred under argon atmosphere for 2.5 h under 50 °C. After that, the reaction mixture was quenched with potassium sodium tartrate solution (5.0 mL). Most organic solvents were removed under reduced pressure and the mixture was extracted with *i*-PrOH:$CHCl_3$ = 1:3 (40.0 mL×3).The combined organic layers were washed with brine (5.0 mL). After dried over Na_2SO_4, the solution was concentrated *in vacuo* to afford the desired compound **7** as a white solid without further purification.

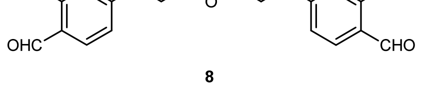

8

Compound **8**: To a solution of compound **7** (56.0 mg, 0.150 mmol) in anhydrous DCM (9.00 mL) was added Dess-Martin periodinane (0.382 g, 0.901 mmol) at room temperature. The resulting mixture was stirred under argon atmosphere for 12 h. After that, the reaction mixture was quenched with Sat. $NaHCO_3$ (5.0 mL) and Sat. $Na_2S_2O_3$ (5.0 mL). The mixture was stirred for 1 h and extracted with DCM (15.0 mL×3). The combined organic layers were washed with brine (5.0 mL). After dried over Na_2SO_4, the solution was concentrated *in vacuo* and purified by silica gel flash chromatography (petrol ether/EtOAc, 3:1 - 3:2) to afford the desired compound **8** as a white solid (51.7 mg, 93% for two steps).

1H NMR (500 MHz, CDCl3): δ 10.65 (s, 2H), 10.32 (s, 2H), 7.91 (d, J = 8.0 Hz, 2H), 7.46 (d, J

= 2.0 Hz, 2H), 7.23 (dd, J = 8.0, 2.0 Hz, 2H), 4.30 (m, 4H), 3.98 (m, 4H); 13C NMR (125 MHz, CDCl3): δ 191.9, 191.1, 163.1, 138.8, 134.8, 129.9, 119.5, 115.3, 69.8, 68.3; HRMS (m/z) calcd. for C20H19O7, 371.1125; found, 371.1123.

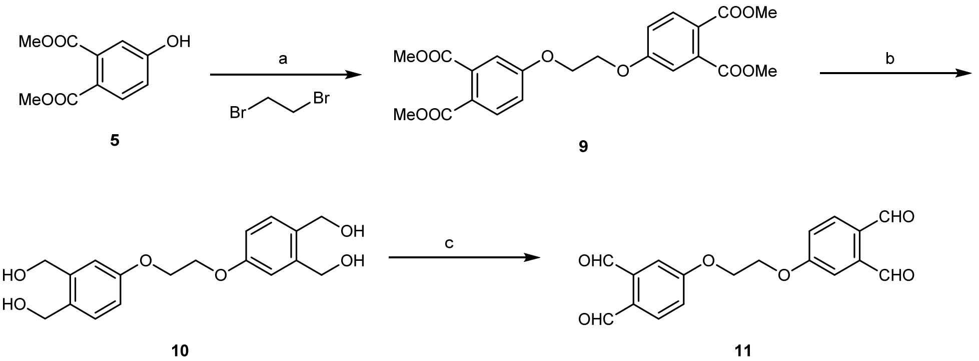

Reagents and conditions: (a) Cs_2CO_3, DMF, 80 °C, 12 h, 37%; (b) DIBAL-H, THF, 12 h; (c) Dess-Martin periodinane, DCM, 6 h, 80% for two steps.

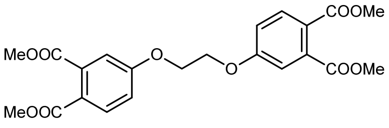

9

Compound **9**: To a solution of Cs_2CO_3 (6.20 g, 19.0 mmol) and compound **5** (2.10 g, 10.0 mmol) in anhydrous DMF (100.0 mL) was added ethylene dibromide (7.10 g, 38.0 mmol) at 80 °C. The resulting mixture was stirred under argon atmosphere for 12h. After that, the reaction mixture was filtered to remove the solid then the filtrate was concentrated *in vacuo* and purified by silica gel flash chromatography (petrol ether/EtOAc, 10:1 - 5:1) to afford the desired compound **9** as a white solid. If anyone wants to improve the yield of this step, you can decrease the ratio of dibromide (1.60 g, 37%).

1H NMR (400 MHz, CDCl3): δ 7.81 (d, J = 8.0 Hz, 2H), 7.12 (d, J = 2.0 Hz, 2H), 7.03 (dd, J = 8.0, 2.0 Hz, 2H), 4.39 (s, 4H), 3.89 (d, J = 16.0 Hz, 12H); 13C NMR (100 MHz, CDCl3): δ 168.7, 166.9, 161.0, 135.8, 131.7, 123.0, 116.6, 114.2, 66.7, 52.9, 52.6; HRMS (m/z) calcd. for C22H23O10, 447.1286; found, 447.1282.

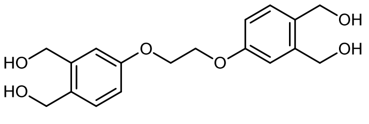

10

Compound **10**: To a solution of compound **9** (0.717 g, 1.61 mmol) in anhydrous THF (40.0 mL) was added DIBAL-H (16.1 mL, 16.1 mmol, 1.0 M in hexanes) at room temperature. The resulting mixture was stirred under argon atmosphere for 12 h. After that, the reaction mixture was quenched with potassium sodium tartrate solution (25.0 mL). Most organic solvents were removed under reduced pressure and the mixture was extracted with *i*-PrOH:$CHCl_3$ = 1:3 (100.0 mL×3).The combined organic layers were washed with brine (50.0 mL). After dried over Na_2SO_4, the solution was concentrated *in vacuo* to afford the desired compound **10** as a white solid without further purification.

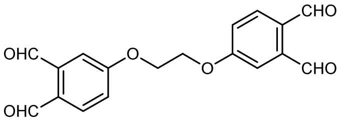

11

Compound **11**: To a solution of compound **10** (0.150 g, 0.450 mmol) in anhydrous DCM (40.0 mL) was added Dess-Martin periodinane (1.10 g, 2.69 mmol) at room temperature. The resulting mixture was stirred under argon atmosphere for 6 h. After that, the reaction mixture was quenched with Sat. $NaHCO_3$ (20.0 mL) and Sat. $Na_2S_2O_3$ (20.0 mL). The mixture was stirred for 1 h and extracted with DCM (40.0 mL×3). The combined organic layers were washed with brine (30.0 mL). After dried over Na_2SO_4, the solution was concentrated *in vacuo* and purified by silica gel flash chromatography (petrol ether/EtOAc, 5:1 - 1:2) to afford the desired compound **11** as a

white solid (0.119 g, 80% for two steps).

1H NMR (400 MHz, $CDCl_3$): δ 10.69 (s, 2H), 10.34 (s, 2H), 7.96 (d, J = 8.0 Hz, 2H), 7.53 (d, J = 2.0 Hz, 2H), 7.29 (dd, J = 8.0, 2.0 Hz, 2H), 4.53 (s, 4H); 13C NMR (100 MHz, $CDCl_3$): δ 191.8, 191.1, 162.7, 138.8, 134.9, 130.2, 119.7, 115.0, 66.9; HRMS (m/z) calcd. for $C_{18}H_{15}O_6$, 327.0863; found, 327.0855.
