# Supplementary material for: Characterization of protein unfolding by fast cross-linking mass spectrometry using di-ortho-phthalaldehyde cross-linkers
Source: Nat Commun. 2022 Mar 18;13:1468. doi: 10.1038/s41467-022-28879-4 (PMC8933431; doi:10.1038/s41467-022-28879-4)
Supplement: Supplementary file 3 — Description of Additional Supplementary Files [file 41467_2022_28879_MOESM3_ESM.docx]

**Description of Supplementary Files**

**File Name:** Supplementary Data 1

**Description:** Annotation of the reaction products of OPA with each of ten synthetic peptides in the selectivity test.

**File Name:** Supplementary Data 2

**Description:** The initial and final conformations and four categories of simulated structures of RNase A in 8 M urea.

**File Name:** Supplementary Movie 1

**Description:** Animated snapshots of the unfolding process of RNase A.
