# Supplementary material for: Characterization of protein unfolding by fast cross-linking mass spectrometry using di-ortho-phthalaldehyde cross-linkers
Source: Nat Commun. 2022 Mar 18;13:1468. doi: 10.1038/s41467-022-28879-4 (PMC8933431; doi:10.1038/s41467-022-28879-4)

(a - j) MS1 chromatographic peaks and the corresponding HCD spectrum. Lysine ε-NH_2_ or peptide N-terminal α-NH_2_ reacted with OPA, forming N-substituted phthalimidines (product 1, Δ mass = + 116.0262 Da). The loop-linked side product (product 2, Δ mass = +98.0156 Da) resulted from OPA joining an amino group and another nucleophilic group on the same peptide. The modified residues are indicated in red. Notes: peptide GR-11 (in d) has no loop-linked side products due to dimethylation of its N-terminus; the sixth peak in peptide VK-9 (in g) was a mixture of two products, as labelled in MS2 spectrum; the second and fourth peaks in peptide HR-9 (in i) were presumed to contain the loop-linked side products between N terminal α-NH_2_ and a sulfhydryl group from cysteine, but some fragment ions support the addition of 98.0156 Da at the N terminus without clear explanation, as labelled in MS2 spectrum.


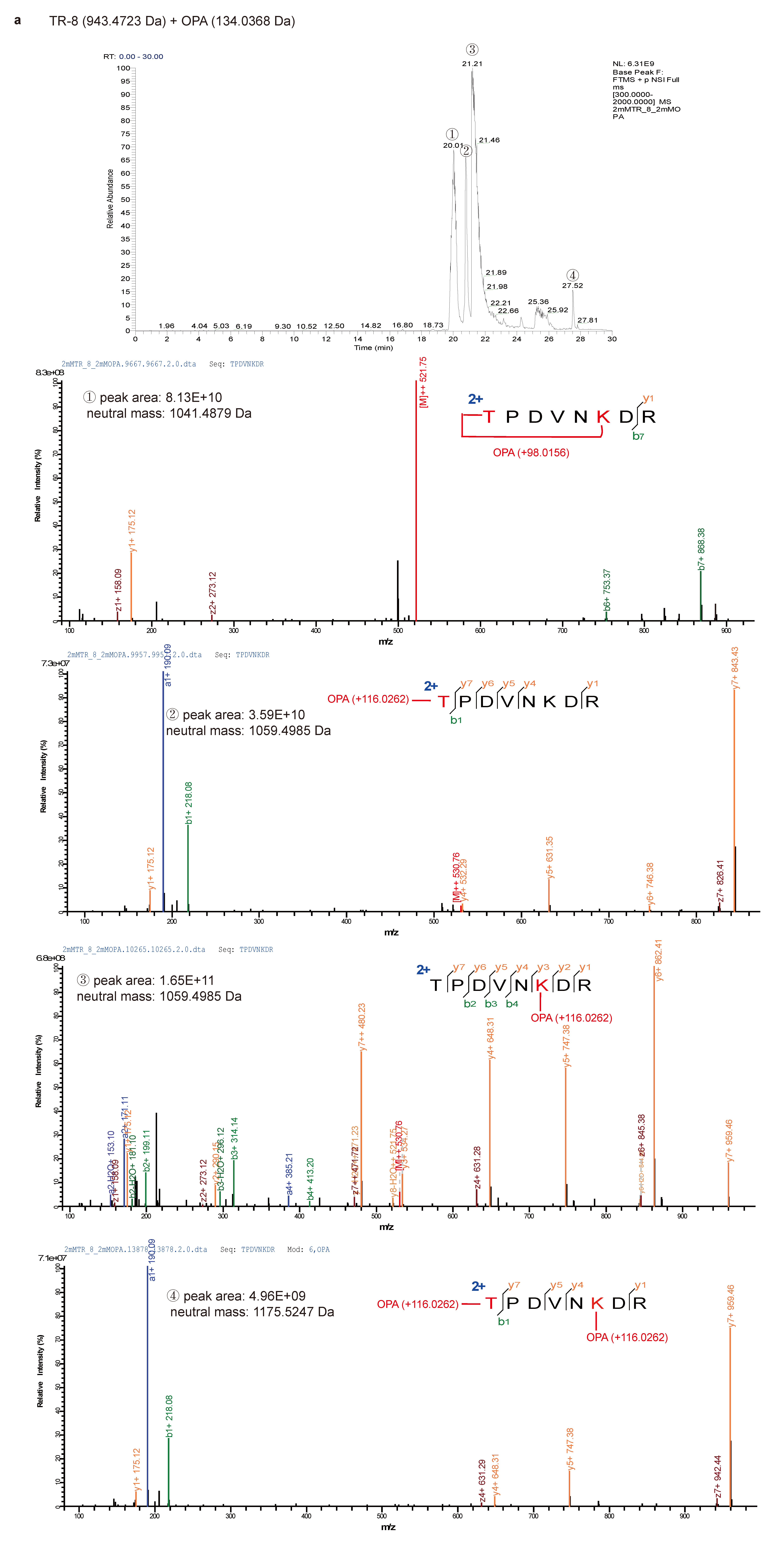


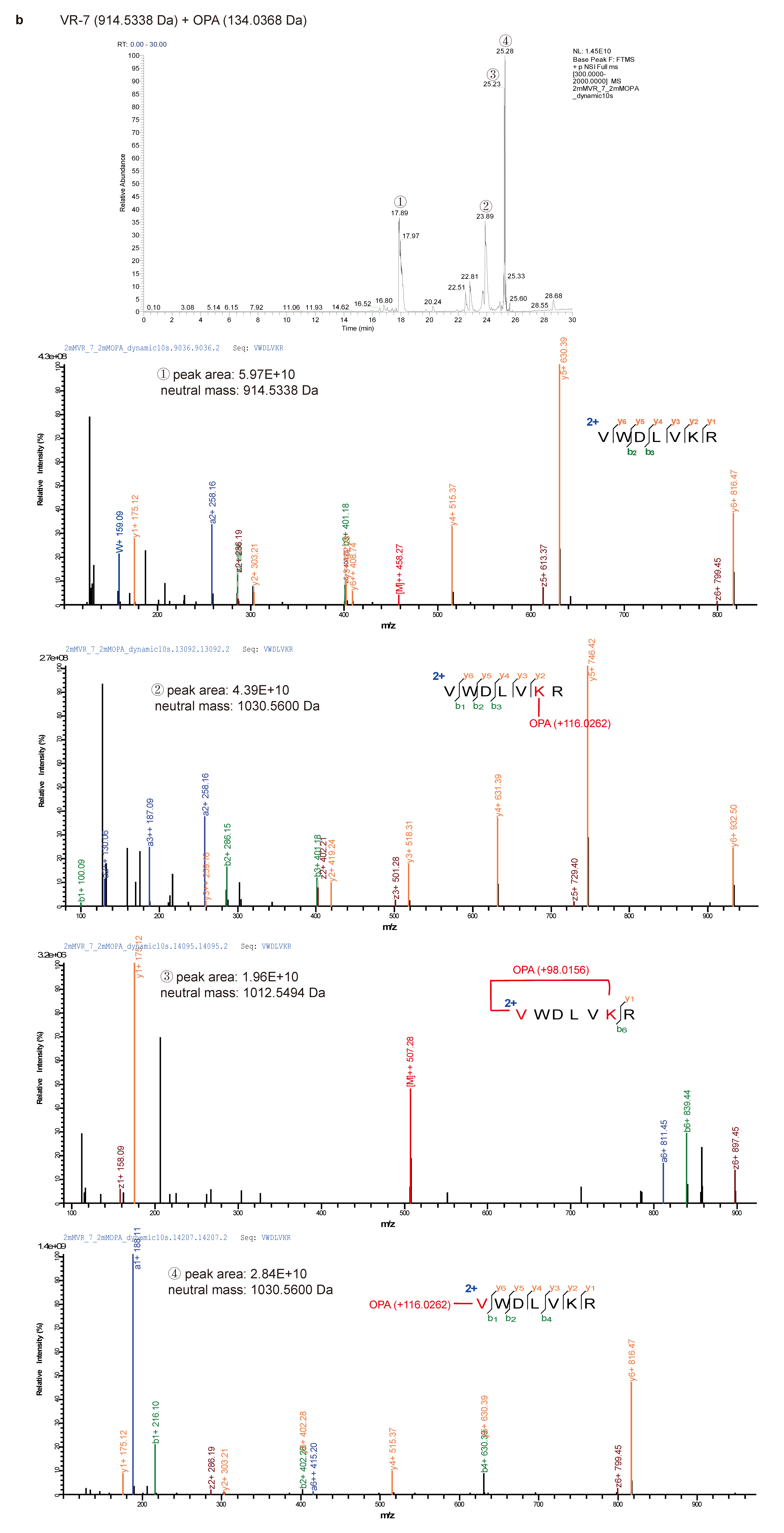


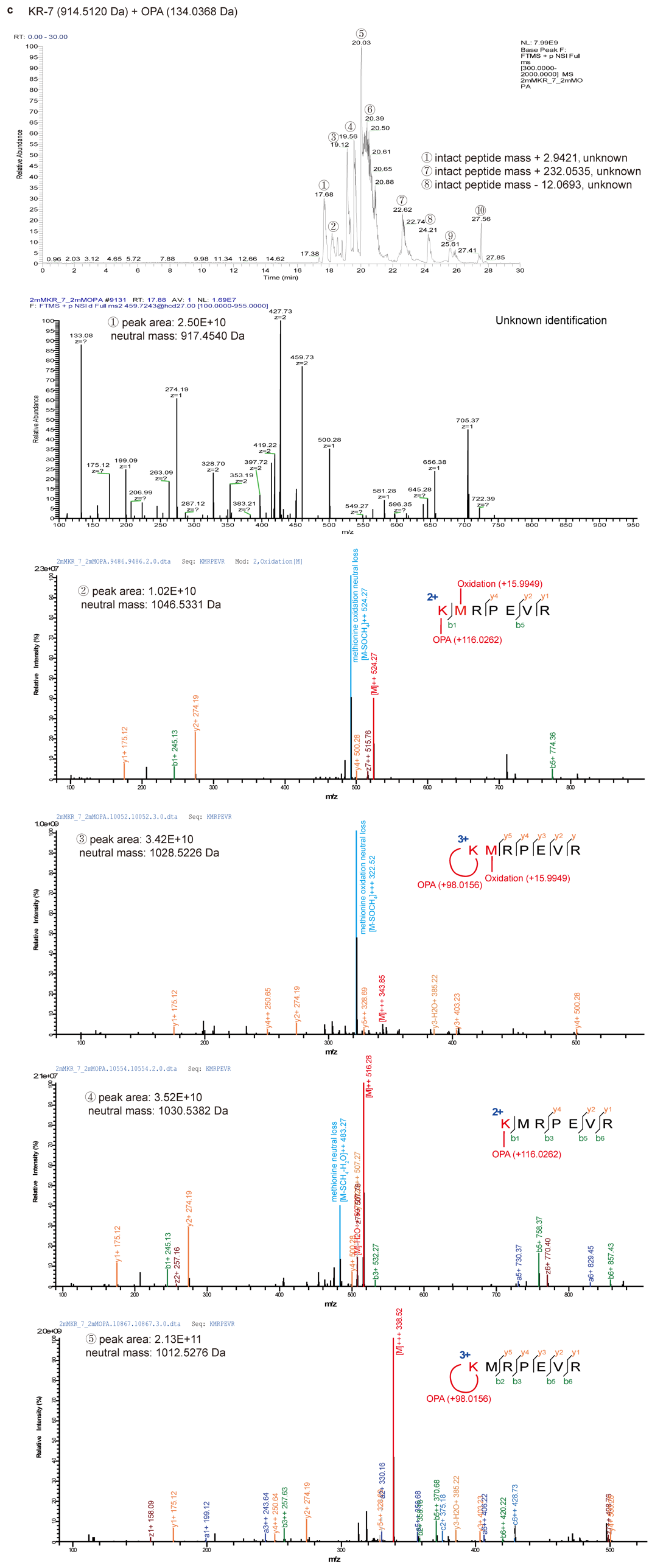


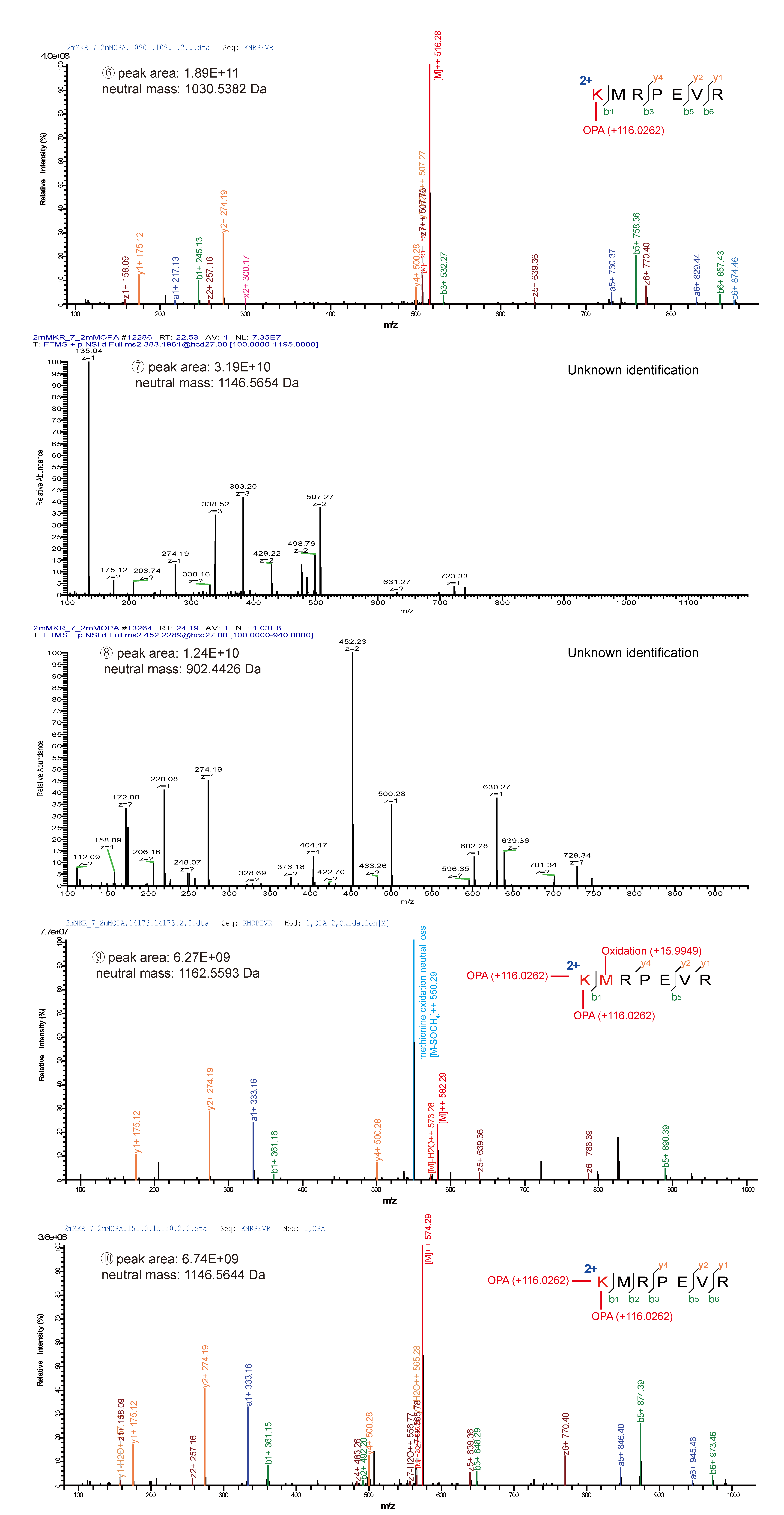


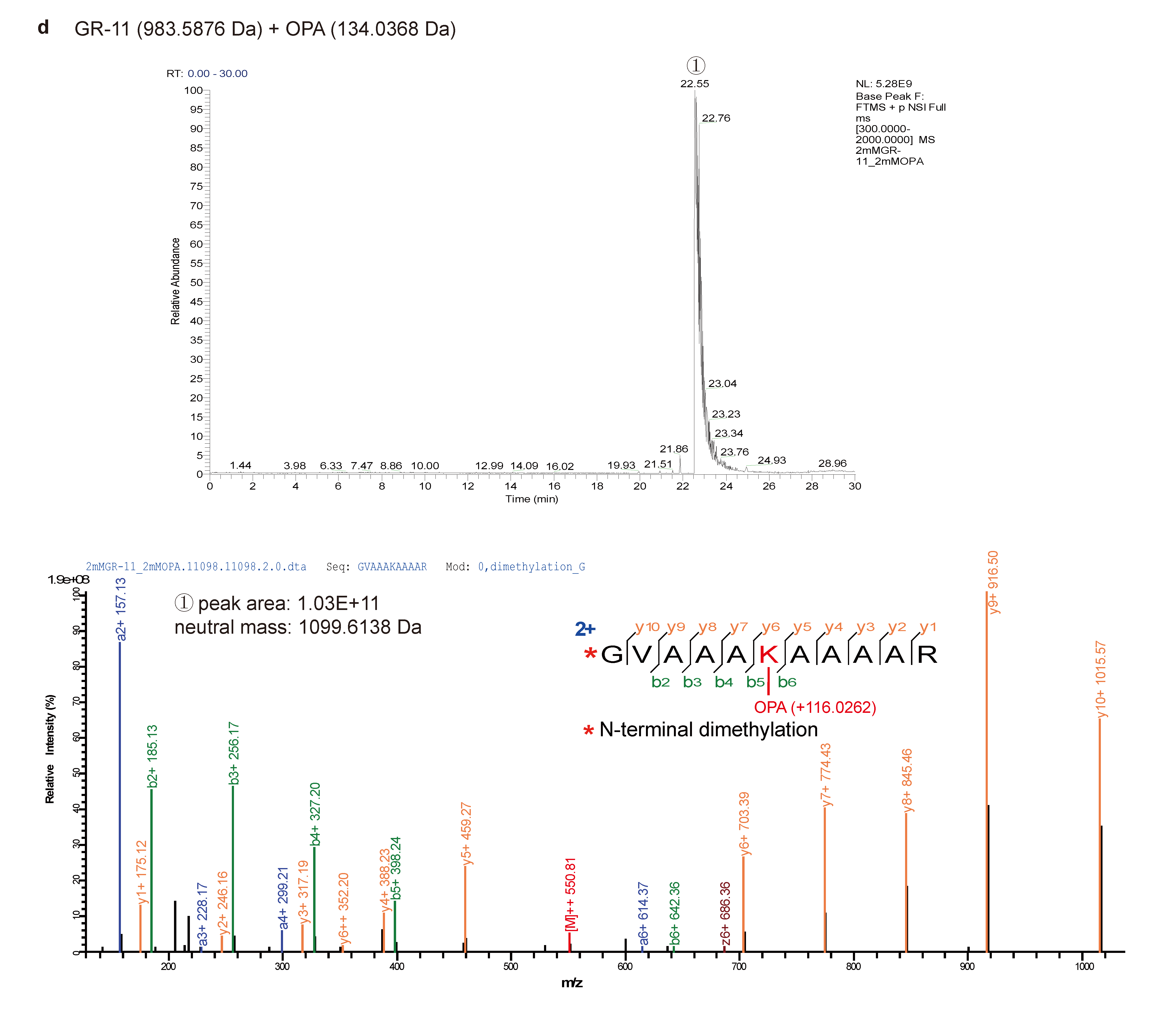


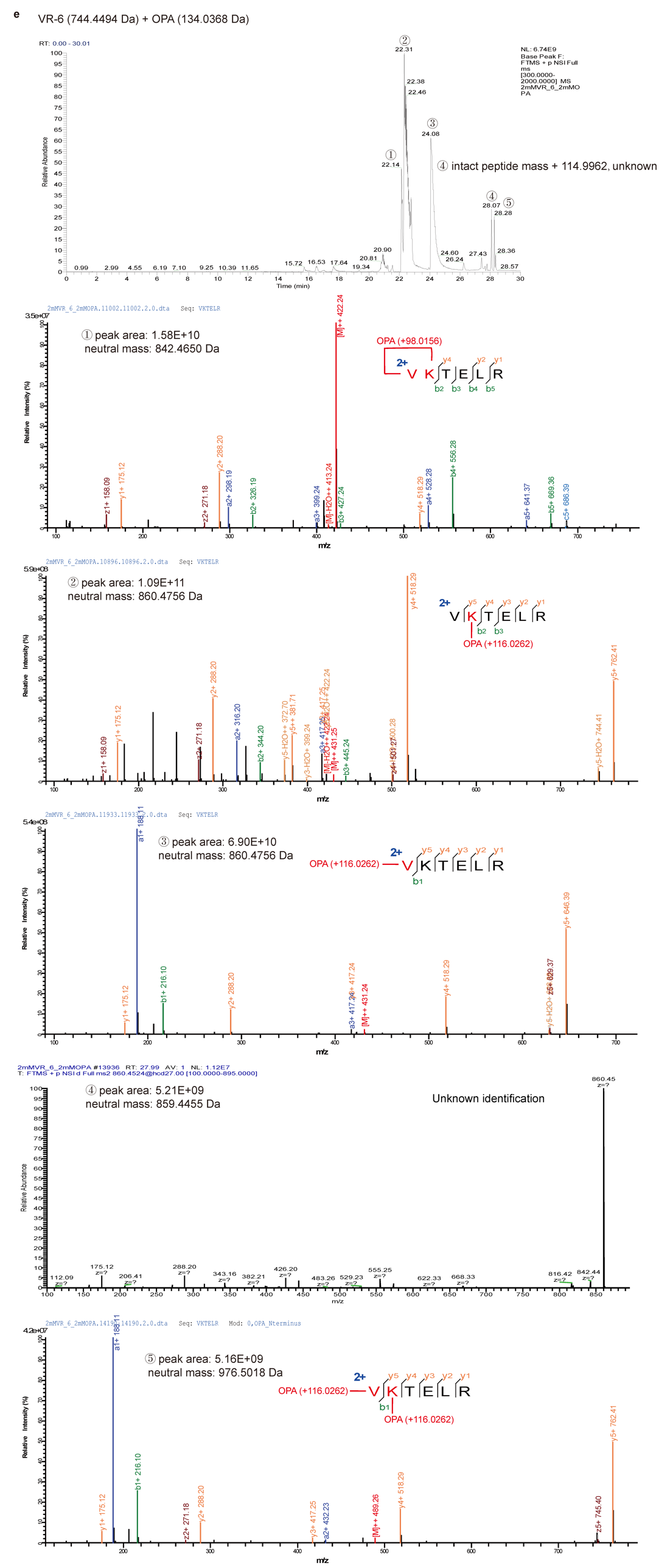


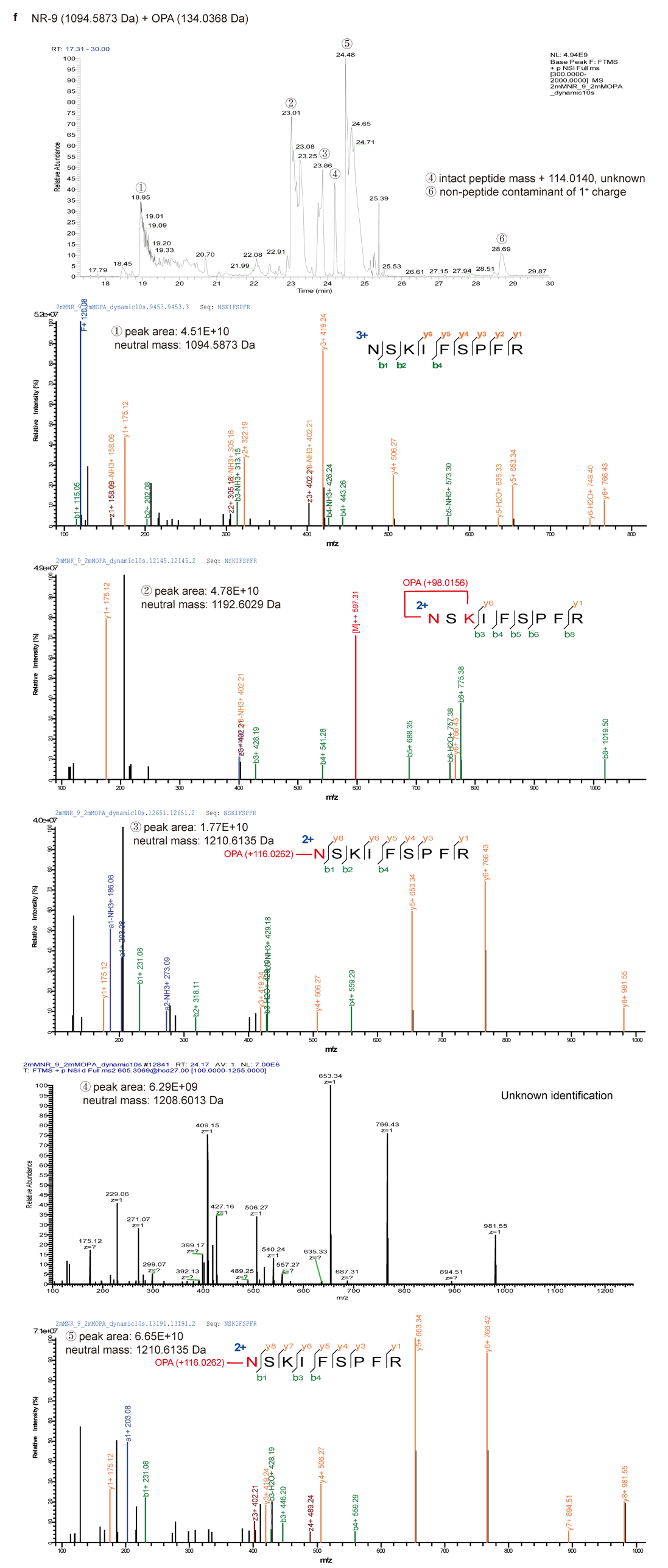


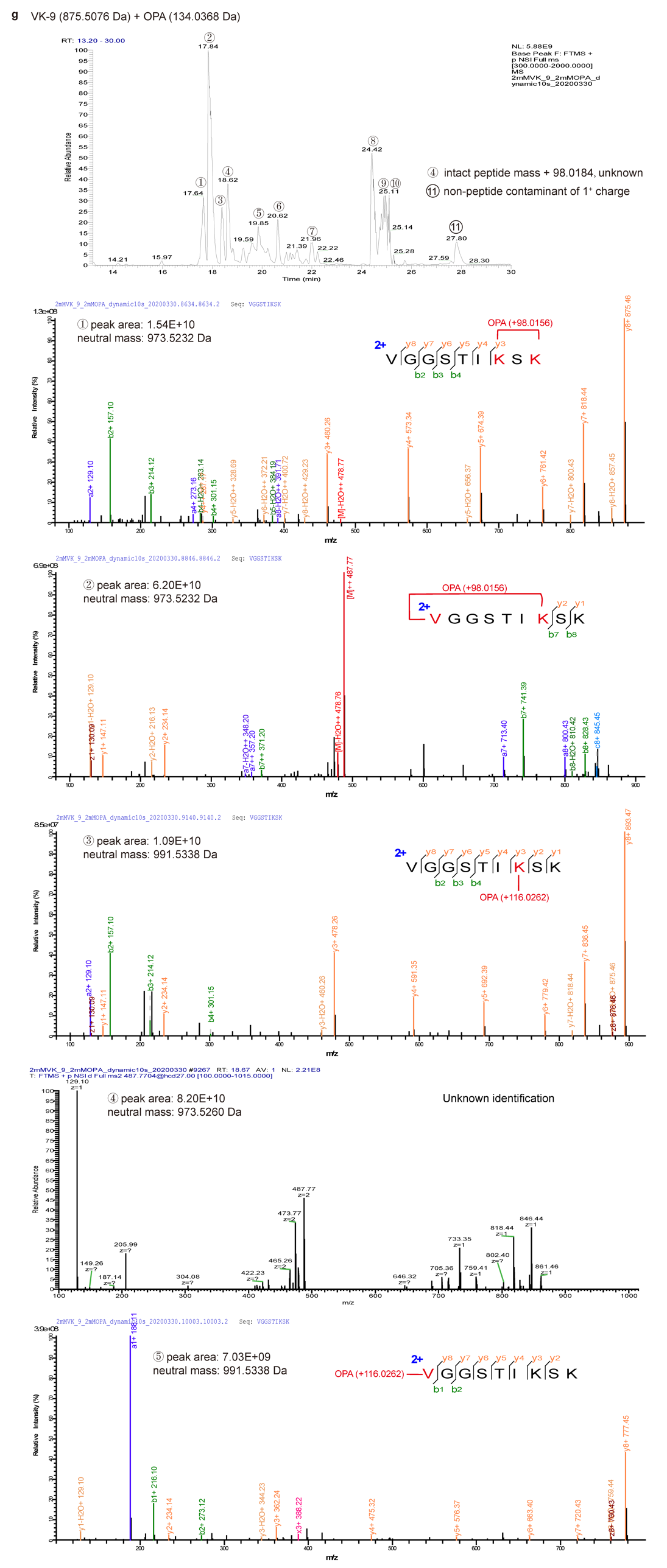


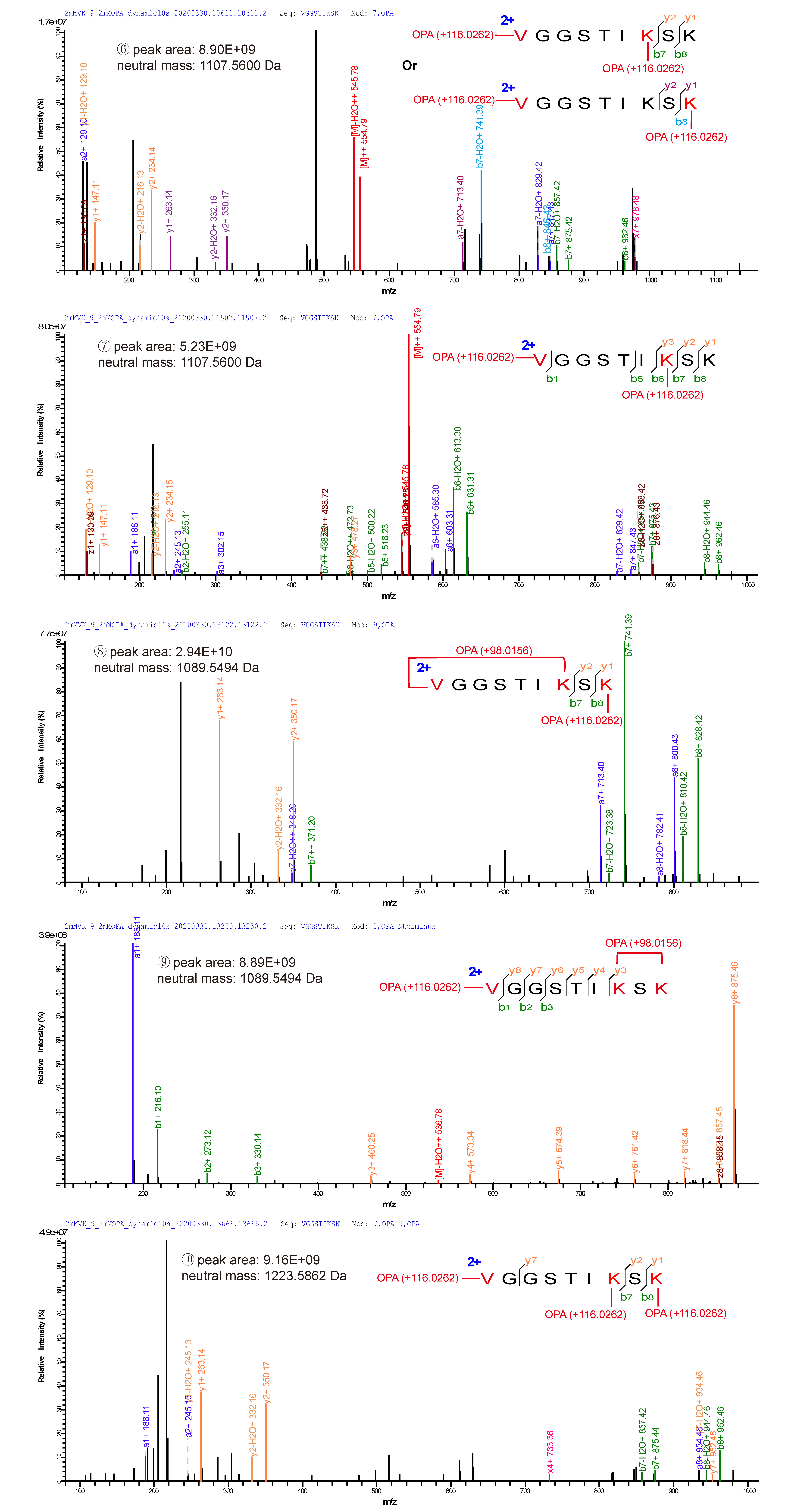


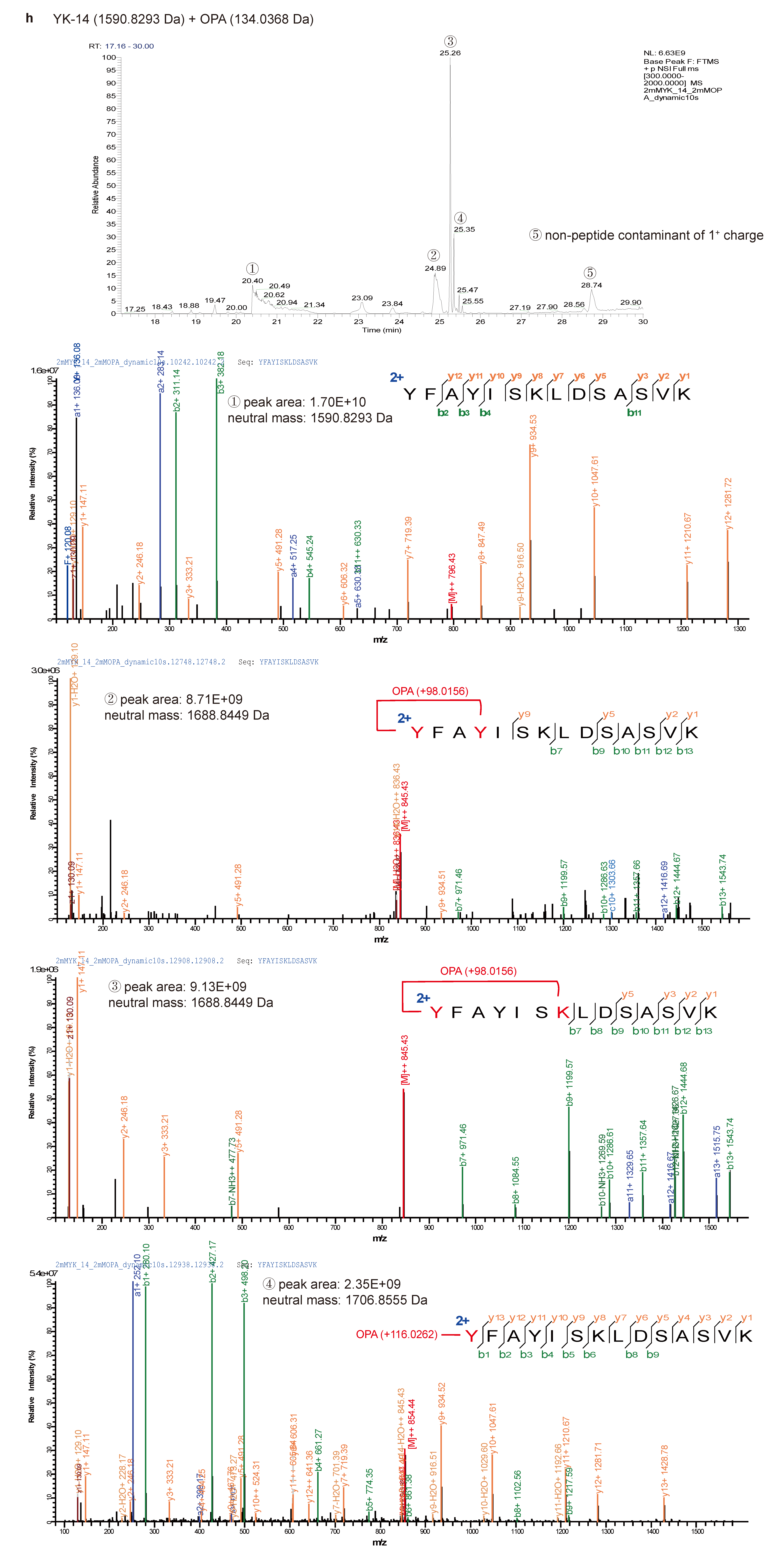


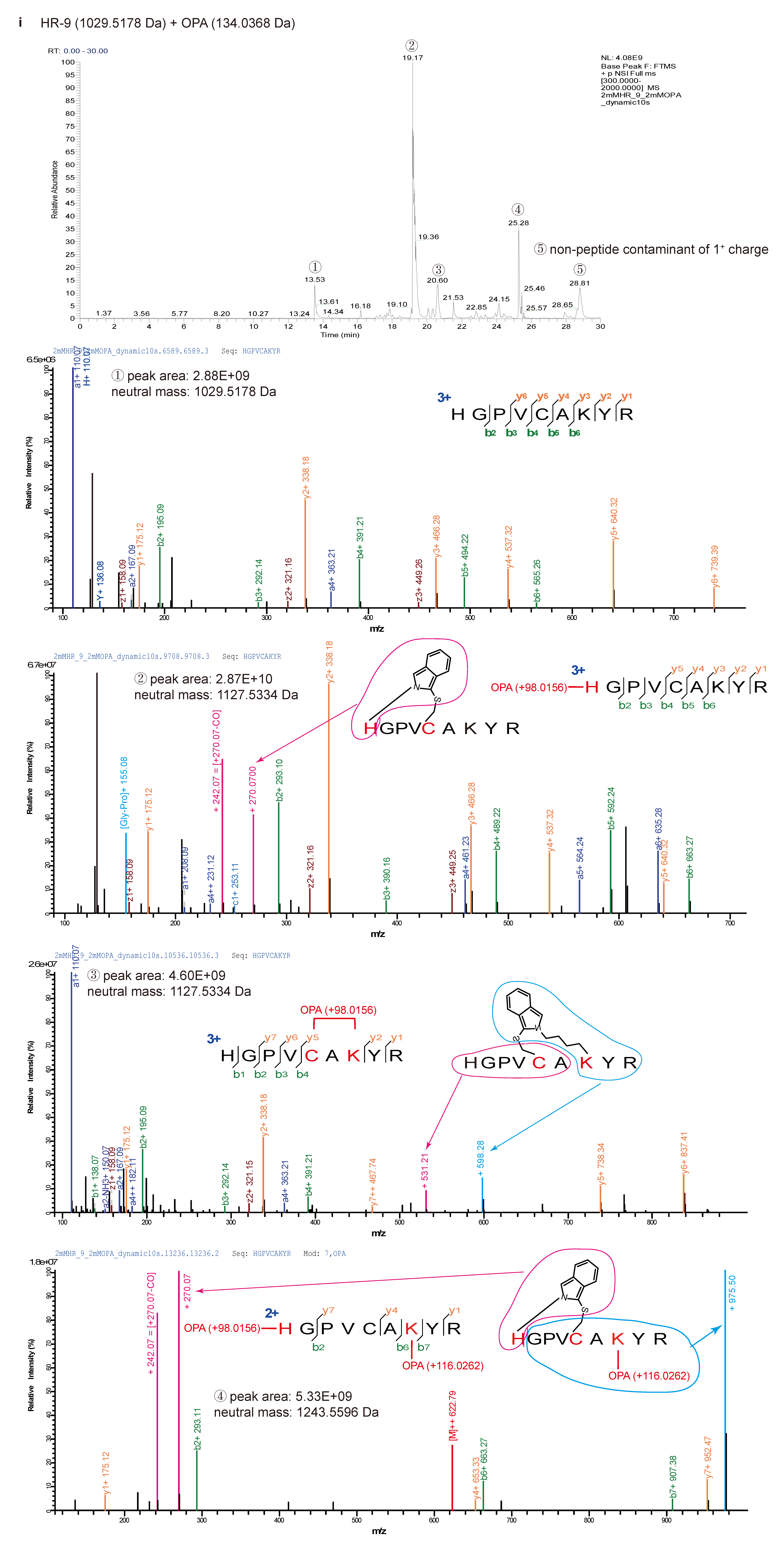


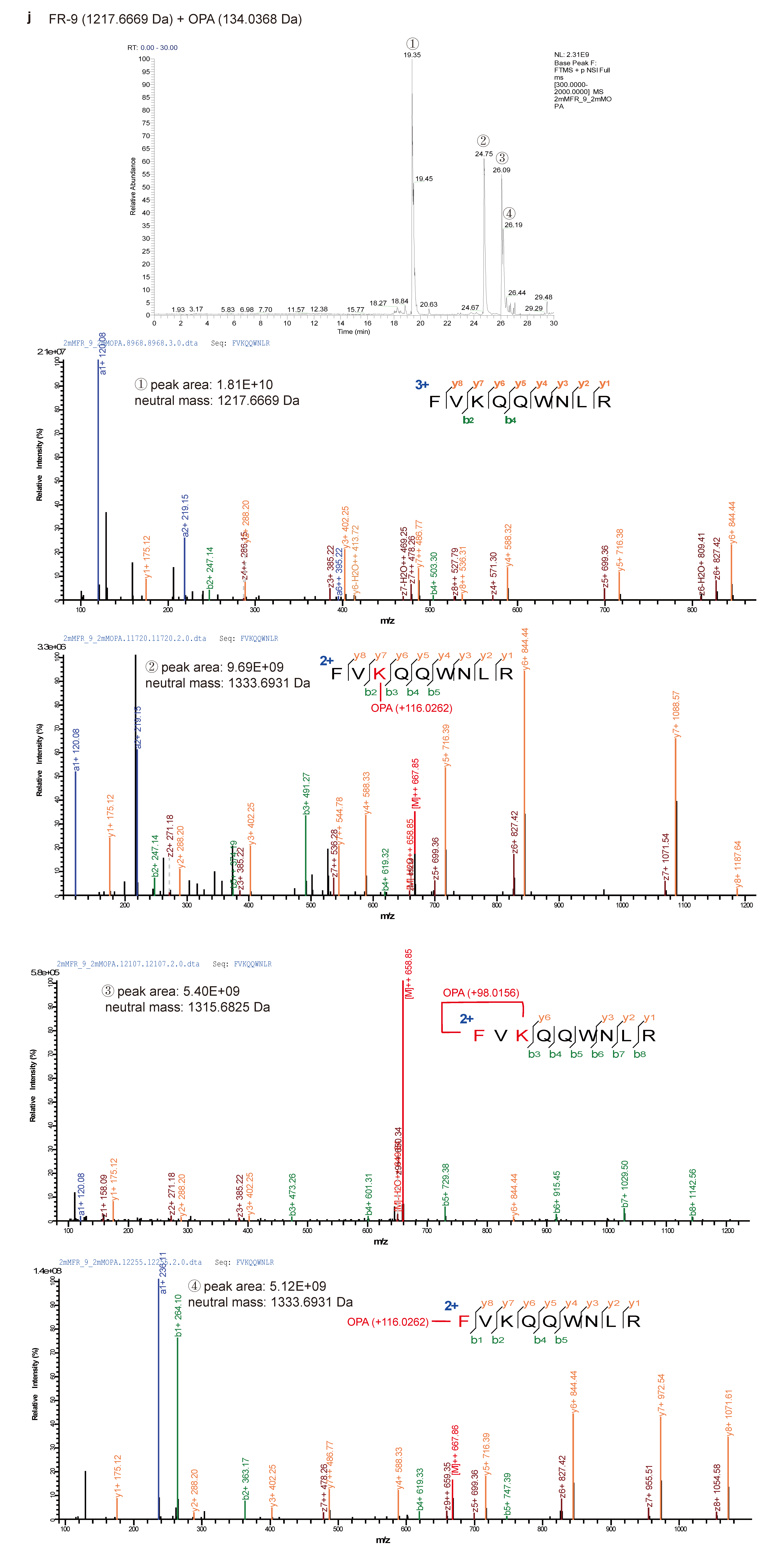

Supplement: Supplementary file 4 — Supplementary Data 1 [file 41467_2022_28879_MOESM4_ESM.docx]
